# Probing Functional Properties of Nociceptive Axons Using a Microfluidic Culture System

**DOI:** 10.1371/journal.pone.0080722

**Published:** 2013-11-20

**Authors:** Christoforos Tsantoulas, Clare Farmer, Patricia Machado, Katsuhiro Baba, Stephen B. McMahon, Ramin Raouf

**Affiliations:** 1 Pfizer KCL Pain Labs, Wolfson Centre for Age-Related Diseases, King’s College London, London, United Kingdom; 2 Pfizer Global R&D, Sandwich, United Kingdom; 3 Wolfson Centre for Age-Related Diseases, King’s College London, London, United Kingdom; Stanford University School of Medicine, United States of America

## Abstract

Pathological changes in axonal function are integral features of many neurological disorders, yet our knowledge of the molecular basis of axonal dysfunction remains limited. Microfluidic chambers (MFCs) can provide unique insight into the axonal compartment independent of the soma. Here we demonstrate how an MFC based cell culture system can be readily adapted for the study of axonal function *in vitro*. We illustrate the ease and versatility to assay electrogenesis and conduction of action potentials (APs) in naïve, damaged or sensitized DRG axons using calcium imaging at the soma for pharmacological screening or patch-clamp electrophysiology for detailed biophysical characterisation. To demonstrate the adaptability of the system, we report by way of example functional changes in nociceptor axons following sensitization by neurotrophins and axotomy *in vitro*. We show that NGF can locally sensitize axonal responses to capsaicin, independent of the soma. Axotomizing neurons in MFC results in a significant increase in the proportion of neurons that respond to axonal stimulation, and interestingly leads to accumulation of Nav1.8 channels in regenerating axons. Axotomy also augmented AP amplitude following axotomy and altered activation thresholds in a subpopulation of regenerating axons. We further show how the system can readily be used to study modulation of axonal function by non-neuronal cells such as keratinocytes. Hence we describe a novel *in vitro* platform for the study of axonal function and a surrogate model for nerve injury and sensitization.

## Introduction

The ability of axons to reliably initiate and propagate APs over long distances is central to the functioning of the nervous system. Increasing evidence suggests that axons not only participate in a significant degree of signal processing within the nervous system [1,2] but are also able to control the local expression of ion channels and signal transduction molecules through the axonal mRNA translation machinery, independent of the soma [3]. This autonomy from the somal compartment is particularly relevant in the peripheral nervous system, where sensory axons have the added task of ‘sensing’ stimuli, innocuous or noxious, and to relay the information to the spinal cord. Since many neurological disorders, such as neuropathic pain, involve degeneration or maladaptive changes in axonal function (e.g. hyperexcitability), a detailed understanding of the molecular physiology of axons is required. However, technical limitations have slowed progress in this area although a number of methodologies have been devised, e.g. the skin-nerve or the excised DRG preparation [4,5]. The majority of these techniques rely on *in vivo* preparations that are often difficult to maintain and, more importantly, are not readily amenable to genetic or pharmacological manipulations. 

Establishing methodologies to study axonal function *in vitro* in a specific and controlled manner has been challenging. Traditional neuronal cultures intrinsically rely on characterization of cell soma responses as the principle outcome measure and do not allow selective axonal manipulations. Therefore, dissociation of the two compartments and evaluation of the axonal responses (generation and propagation of APs) specifically would be ambiguous. Similarly, attempts to characterize trafficking of protein complexes in the axons lack the appropriate spatial resolution. Furthermore, these preparations are typically under the constant influence of supportive cells such as fibroblasts and Schwann cells, which may affect axonal responses. Advances in microfluidics technology have provided a way to overcome these restrictions [6]. These devices facilitate the separation of the cell body from associated processes in distinct compartments, and thus allow independent characterization of these components. 

Peripheral hyperexcitability of DRG axons, which is a contributing cause of neuropathic pain conditions, can result from diverse regulatory processes and is primarily associated with injury-induced modifications in expression and function of ion channels and receptors [7]. It is now known that the constitutive or regulated trafficking of these constituents can significantly affect their function, and therefore a plethora of studies are focused on changes in subcellular localization and axonal transport that occur in pain syndromes [8]. For instance, it has been documented that human neuromas become hyperexcitable [9–11] which may result from accumulation of ion channels. Pro-nociceptive NGF and other mediators have been shown to increase TRPV1 incorporation to the cell membrane and anterograde transport [12–15], while other pain-related channels like P_2_X_3_ and TRPA1 are subject to similar modulatory processes [16–18]. In addition, interactions of axons with non-neuronal cells have been proposed to further modulate pain signalling in the peripheral nervous system [19,20]. In this context, understanding the underlying mechanisms that lead to changes in axonal function would greatly benefit from tissue culture models that can recapitulate salient aspects of axonal function *in vivo*. 

Here, we have optimized and adapted a microfluidics based culture system [21,22] that allows isolation of DRG axons and can be combined with a variety of imaging, biochemical and functional manipulations. We used this system to assay the ability of axons to generate and propagate APs to the soma, hence recapitulating the *in vivo* function of the axons. We also demonstrate the use of the MFC in investigating changes in axonal excitability following sensitization by nerve growth factor (NGF), injury by axotomy, and interaction with keratinocytes. Thus we describe how axonal properties can be readily interrogated using a combination of pharmacological and molecular imaging tools in the MFC culture system.

## Materials and Methods

### Animals

Male C57BL/6 mice and pregnant Wistar rats were purchased from Charles-River UK. All animals were maintained in a designated facility in strict accordance to the UK Home Office Code of Practice for the Housing and Care of Animals Used in Scientific Procedures. Animals were sacrificed and tissue was collected in strict accordance to the UK Home Office regulations and procedures under Schedule 1 of Animals (Scientific Procedures) Act 1986. All procedures were approved by the Animal Welfare and Ethical Review Body at King’s College London (project licence PPL 70/6868).

### Preparation of DRG cultures in microfluidic chambers (MFC)

Pre-sterilized 40 mm-diameter glass bottom dishes (WillCo Wells) were coated with 0.01 mg/ml poly-L-Lysine followed by 20 μg/ml laminin for 2 hrs at 37°C. Microfluidic devices (Xona Microfluidics) were prepared, attached to the glass plates and seeded using a modified version of the manufacturer’s protocol (Methods S1 in File S1). The SND450 design (microgroove length of 450 μm) was chosen for neonate rat MFC, while for adult mouse cultures the SND150 or SND450 options were used. In the experiments utilizing the triple compartment design, we used the TCND500 model (two 500 μm microgroove barriers with a 500 μm central chamber). 

Dissociated primary neuron cultures were prepared from naive neonate rats (P1-P4) or adult mice (15-20 g), as described before [5]. Briefly, DRGs from all segmental levels were aseptically excised and microdissected to remove roots and expose DRG capsules. Following this, the ganglia were digested with enzyme mixture (0.1% collagenase type XI, 3 mg/ml dispase, 10 mM glucose, 5mM HEPES) for 30 min in a 37°C and 5%CO_2_ humidified incubator. DRGs were washed in Hank's Balanced Salt Solution (HBSS, Invitrogen), followed by gentle mechanical dissociation in 1 ml of solution, using a Gilson P1000 pipette. Triturated cells were pelleted by centrifugation (58 g for 6 min) through a 15% bovine serum albumin (BSA) cushion, prepared by mixing 1 ml of HBSS with 1 ml 30% BSA Fraction V solution (Sigma). Finally, neuronal pellets were resuspended in 10-20 μl of neurobasal growth medium (Gibco), supplemented with 2% B27 supplement (Invitrogen), 1% Glutamax, 100 units/ml penicillin and 100 µg/ml streptomycin. 

Approximately 4-6 x 10^5^ neurons were plated in the microfluidic chambers, as described in the methods S1 in File S1. Cultures were maintained at 37°C in a humidified 5% CO_2_ incubator. In order to establish a neurotrophin gradient to facilitate axonal growth through microgrooves, media was supplemented with mouse nerve growth factor (mNGF, Alomone) and human glial cell line-derived neutotrophic factor (hGDNF, Alomone) as follows; Day 0-1: 100 ng/ml of each neurotrophin; Day 1-3: somal compartment, 50 ng/ml; axonal compartment, 100 ng/ml; Day 3 onwards: somal compartment, 25 ng/ml; axonal compartment, 100 ng/ml and replaced every 48 hrs thereafter.

### Calcium imaging and analysis

After 5 days in culture, the fluorescent tracer DiO (Invitrogen, 1/200) was added in the axonal compartment and allowed to be taken up overnight by crossing neurons. The following day, neurons in the somal compartment were loaded with 2 μM of the calcium indicator dye Fura-2-AM (Invitrogen), 1mM probenecid (Sigma) and 0.02% pluronic acid (Invitrogen) in imaging buffer consisting of Ca^2+^ and Mg^2+^ -free HBSS, supplemented with 1 mM MgCl_2_, 1 mM CaCl_2_, 10 mM HEPES (Gibco), for 45 min at 37°C. Both MFC compartments were washed twice with imaging buffer before the assay commenced. Microfluidic chambers were mounted on an inverted microscope connected to an EasyRatioPro imaging system (Photon Technology International, UK), comprising of a Quantum CCD camera (Photometrics, USA) and analysis software (EasyRatio Pro) capable of acquiring digitized images every 500 ms upon illumination with the appropriate light source. First, bright field and DiO staining were visualised and only neurons exhibiting staining in the soma (indicative of axonal crossing) were chosen for further analysis. Areas of interest (ROI) were drawn around the corresponding cell soma and fluorescent images at 510 nm were collected at 2 Hz using 340 and 380 nm excitation wavelengths. The 510 nm fluorescence intensity F_340_/F_380_ ratio (F_ratio_) was plotted against time for each ROI. Unless stated otherwise, cells were stimulated with 100 nM capsaicin (diluted 1/1000 from a DMSO stock, Sigma), or 30 mM KCl in imaging buffer. Axonal responses were always acquired first, while somal stimulation was applied at the end of the assay to avoid desensitization or apoptotic cell death due to larger Ca^2+^ influx upon direct somal stimulation. Each drug application was separated by a wash in imaging buffer followed by a 10 min rest period. In the lidocaine experiment, the drug was perfused through the middle compartment 5 min before recordings commenced. Throughout the assay, caution was taken to retain the fluidic isolation between somal and axonal compartments (i.e. for axonal stimulation volume was higher on the somal compartment and this was reversed for somal stimulation). A positive response was assigned to a neuron when a maximum increase in F_ratio_ greater than 3 times the standard deviation above the baseline (calculated from at least 10 s prior to drug application) was observed following drug application. The maximum increase (ΔF_ratio_) was defined as the difference between peak response and baseline. All positive responses and peaks were confirmed by visual inspection of the ratio plots. The somal KCl response was regarded as the maximal Ca^2+^ signal and was used to normalise data between different experiments. Dose response curves and the EC_50_ values were calculated by fitting a three parameter logistic equation (Y=100/(1+10^(LogEC_50_-X)) to the data, for both ΔF_ratio_ and % responders against agonist concentration plots, using GraphPad prism software.

Student t-test (unpaired, equal variance) was used to compare groups and results are reported as mean ± SEM, where n = number of microfluidic cultures or cells for each group as appropriate. Statistical significance was set at p < 0.05. 

### *In vitro* axotomy

For *in vitro* axotomy of crossing neurons in MFC, mouse DRG cultures were grown for 72 hrs according to the standard protocol outlined above. At this point*, in vitro* axotomy was carried out by applying suction with a fine tip glass pipette positioned at the entrance of the axonal channel, carefully done to avoid disruption of the chamber-to-glass seal. Suction was retained for approximately 5 s or until all visible liquid in the axonal compartment was removed. The axonal compartment was then washed with media by placing the tip at the channel entrance and vigorously pipetting up and down to remove all detached axons and debris. Following this step, fresh medium was added to the compartment and the MFC was inspected under the microscope to validate axotomy and ensure the integrity of the MFC to glass seal. The media in control cultures were replaced with fresh medium on the same day. In each experiment both axotomized and control MFC cultures were kept for an additional 3 days before being assayed.

### NGF sensitization of axons

For NGF sensitization, cultures were prepared from neonate rats as before, with the exception that GDNF was omitted at all stages from the culture media. At 72 hrs post-plating, NGF containing media were removed from both compartments and replaced by media containing goat anti-NGF (1/1000, Sigma). The cultures were then divided into two groups: the control group was treated with anti-nerve growth factor antibodies until the day of assay (day 6). For cultures to be subjected to chronic NGF sensitization, after 24 hrs the media in axonal compartments were replaced with media containing 100 ng/ml NGF, while the somal compartments were continued in anti-NGF antibodies. The media solution was changed daily post day 3, to minimize the effect of neuronally secreted NGF. After 48 hrs (day 6) the axonal responses were assayed.

### Electrophysiology

Six-well MFCs were used for electrophysiological recordings. To provide micropipette access holes were punched on one side of the chamber using a 5mm biopsy punch tool (Harris, US). Holes were punched close to the microgrooves along the length of the channel thus joining the two wells connected by the channel. This resulted in one large exposed area which could be accessed by the micropipette whilst leaving a small portion of the original channel nearest the microgrooves. Dissociated DRG neurons from adult mice were prepared as described earlier and plated into the remaining channel lip and in the open area next to the microgrooves. Chambers were used for patching after 5-8 days. On the day of recording, DiO was added to the far axonal channel for 1 hr, washed twice and the chamber left for ≥2 hrs to allow for development of the fluorescent signal. 

For current clamp recordings, cell soma and axons were bathed in an extracellular solution of HBSS with 10 mM HEPES, 2 mM CaCl_2_, 1 mM MgCl_2_, pH 7.3. Silver wire stimulating electrodes attached to a constant current stimulator (NL800, Digitimer) under the control of a pulse generator (Neurolog, Digitimer) were placed in the wells of the far axonal channel. A perfusion system was set up across the middle axonal channel. Micropipettes (1.5-2.5 MΩ) were filled with an intracellular solution containing (mM) 120 K-gluconate, 20 KCl, 1 MgCl_2_, 1 CaCl_2_, 10 EGTA, 10 HEPES, 2 Mg-ATP, pH 7.2 with KOH. Only those cells whose soma showed green fluorescence, and therefore had axons which had crossed into the far axonal channel, were used for recording. Whole cell current clamp recordings were performed at room temperature using an Axopatch200 amplifier and pClamp9 software. Periodic electrical stimulation (28 stimuli of 2 ms duration at 5.6 Hz every 20 s) was applied to the axonal side whilst the somal response was monitored using continuous current clamp recording. Pharmacological compounds were applied through the middle axonal channel while somal responses to axonal stimulation were monitored. All data was analysed using Clampfit 9.0, and further analysis and statistical testing was performed using Microsoft Excel and GraphPad Prism software.

### Keratinocyte and DRG neuron MFC co-cultures

Keratinocyte co-cultures with DRG were prepared from age-matched neonate rats, according to methods previously described [23]. Following dissection, the skin sheet was spread completely flat on a clean culture dish with the dermis side facing down, before being transferred to a new dish containing 0.25% trypsin (no EDTA), where it was allowed to float on the solution surface with the dermis side down. After an overnight incubation at 4°C, skin was drained off excess trypsin, spread on a new dish (epidermis side down) and the dermis was carefully removed with forceps. Next, the epidermis sheet was folded and transferred to a deep vessel containing DMEM medium, minced with small scissors and triturated with a 10 ml pipette. The cell suspension was then transferred to a 15 ml tube, leaving stratum corneum sheets behind. Cells were centrifuged at 150 g for 5 min, the pellet was resuspended in DMEM, filtered through a 100 μm cell strainer and subjected to a new centrifugation. Finally, the keratinocyte pellet was resuspended in KGM-2 serum-free medium (Lonza) and cell density was determined using a haemocytometer. 

For MFC co-cultures, a second layer of coating was added to assist keratinocyte attachment. Thus, following attachment of the device on the pre-coated with poly-lysine and laminin dish, the axonal (keratinocyte) compartment was treated with 10 μg/ml bovine fibronectin (BD Biosciences) and 30 μg/ml type I collagen (Sigma) in HBSS for 30 min at 37°C. Approximately 50,000 keratinocytes in a small volume (<10 μl) were loaded in the axonal compartment, immediately after DRG neurons were added in the somal compartment. Cultures were maintained as normal in a 37°C incubator and 24 hrs post-plating the axonal (keratinocyte) side was rinsed with Mg^2+^ and Ca^2+^ -free PBS to remove differentiated cells. KGM-2 medium was supplemented with appropriate neurotrophins according to the standard protocol and replaced every two days. Growth medium on the somal (DRG) side was as described in previous section and microfluidic flow was always towards the axonal (keratinocyte) side. Control MFC cultures were prepared without keratinocytes, but using keratinocyte coating and medium on the axonal side.

### Immunocytochemistry

DRG cultures in MFC were fixed after recording by incubation in 4% PFA solution (made in 0.1 M PB, pH 7.4) for 20 min at room temperature. In order to ensure fixation of axons contained in microgrooves, microfluidic flow was generated via addition of different volumes in each compartment. Following fixation the chambers were washed three times and stored in PBS at 4°C for at least 30 min. Once chilled, wells were emptied and the microfluidic device was removed with a firm upwards motion using forceps. Following this, antibody solutions were added directly on the exposed neurons fixed on the glass bottom. Incubation with the primary antibody was done for 2 hrs at room temperature, followed by three washes and application of the secondary antibody for an additional 45 min. Primary antibodies used were mouse anti-β3tubulin (1:1000; Molecular Probes), rabbit anti-peripherin (1:2000; Chemicon), mouse anti-Nav1.8 (1:500; UC Davis/NIH NeuroMab Facility, clone N134/12), goat anti-transient receptor potential cation channel subfamily V member 1 (TRPV1, 1:500, P-19, Santa Cruz), rabbit anti-calcitonin gene-related peptide (CGRP, 1:2000, Sigma), mouse anti-neurofilament 200 (NF200, 1 : 500, Sigma), rabbit anti-cytokeratin 5 (CK5, 1:500, Abcam) and mouse anti-cytokeratin 10 (CK10, 1:500, Abcam). Secondary antibodies were donkey anti-mouse Alexa 488 or 594, donkey anti-rabbit AlexaFluor 488 or 594 and donkey anti-goat 594 (all 1:1000). For visualization of isolectin B4 (IB4) binding, biotin-conjugated IB4 (1:500, Sigma) followed by AMCA Avidin D (1:400, VectorLabs) was used, while nuclei were stained using 4', 6-diamidino-2-phenylindole (DAPI, 1:1000, Vector Labs). All antibody dilutions were in phosphate buffered saline (PBS) supplemented with 0.2% Triton-X. After a final wash, dishes were mounted with 20 mm diameter round glass coverslips using Fluorsave mounting solution (CalbioChem) and left to dry before imaging.

### Image analysis

Single images or compositions (mosaics) were acquired using Axiovision software on a fluorescent microscope equipped with appropriate filters and connected to a digital camera, while image analysis was conducted using ImageJ software. To determine percentages of crossing neuronal sub-populations, cell body co-localisation counts of the relevant marker and fluorescent tracer were carried out in three independent neonate rat cultures (at least six images at 20x magnification per culture). For quantification of fluorescent signal, a background subtraction was done for each image, followed by thresholding. Quantification of Nav1.8 immunoreactivity was performed by measuring the integrated signal density in control and axotomized cultures (n = 3 per group, at least four 20x images per culture). These images were acquired so that the microgrooves (100 μm long) occupied the center of the image, with approximately 100 μm of somal and axomal compartment length visible on either side. Quantification in the cell soma was done by drawing circular regions of interest (ROIs) around positive neurons on the left (somal) side, while for measurement of signal in microgrooves a fixed size rectangle selection encompassing the full microgroove length was applied to at least 5 microgrooves per image. Quantification of Nav1.8 signal in axonal compartment was done by measuring the integrated density in the total area of the right (axonal) side. For quantification of axonal TRPV1 staining, a rectangle corresponding to the entire axonal compartment was drawn using a mosaic composite image taken at 20x magnification, and the integrated density was calculated for each group. The same size selection was applied onto each image. In order to quantify total axonal growth in the axotomy and NGF treatment experiments, mosaic compositions (taken at 20x) of the entire chamber from peripherin-immunostained cultures were used (n = 3 per group). Like before, a threshold was applied and the area covered by pixels exceeding this threshold was quantified in the entire axonal compartment as an indication of axonal crossing. Values represent mean values ± SEM, and Student’s t-test (unpaired, equal variance) was used to determine statistical significance. 

## Results

### Developing a Ca^2+^-imaging based assay for the study of axonal responses in MFCs

**Figure 1:**
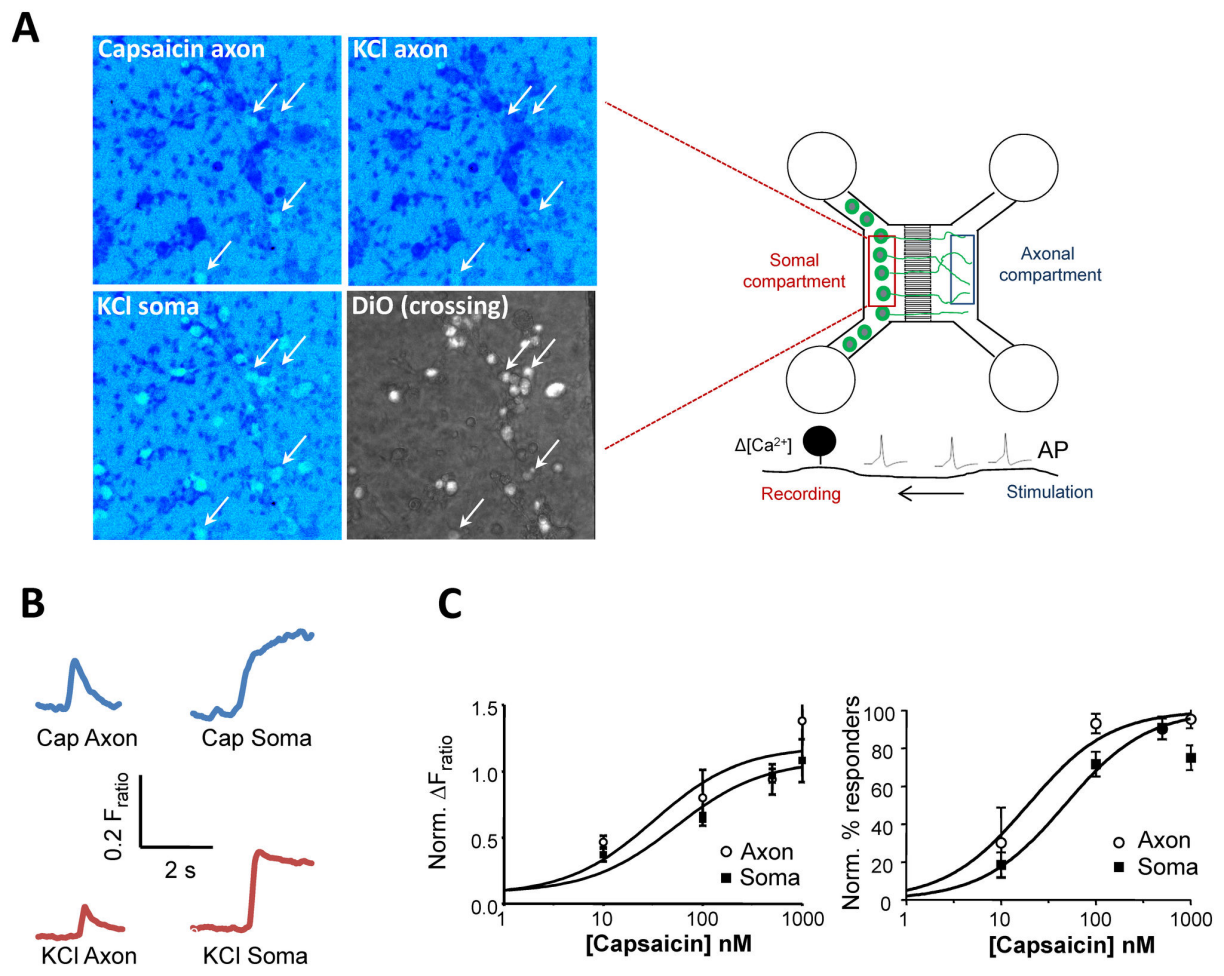
Transduction and transmission of stimuli by axons in MFC. (A) Right, calcium imaging was used to characterize DRG axonal responses in MFC cultures. Schematic shows the assay for axonal function. A stimulus applied to the axonal compartment activates isolated axons and produces an action potential (AP) which propagates along the axon. The subsequent depolarization of the soma membrane facilitates opening of calcium channels, leading to Ca^2+^ influx that can be monitored by the recording setup. Left, representative images of Ca^2+^ responses demonstrating DRG cell soma activation after axonal stimulation with capsaicin (100 nM) or KCl (30 mM). The total number of neurons is determined by responders to KCl applied to the somal compartment (bottom left), while the number of axonal crossings is revealed by the fluorescent tracer uptake (bottom right). (B) Representative traces of Ca^2+^ increases in the neuron soma, following axonal or somal activation with 100 nM capsaicin or 30 mM KCl. (C) Comparison of capsaicin dose-response curves after axonal or somal stimulation. Left, quantification of Ca^2+^ signal; right, percentage of neuronal responders (values represent normalized mean ± SEM, n = 3 per condition).

We first aimed to establish a method to monitor baseline responses and excitability of the isolated axons. Calcium imaging represents a tested and reliable tool to assess neuronal function in culture [24]. Stimulation of DRG axons will result in local depolarization (similar to stimulation of nociceptor nerve endings *in vivo*) and generation of APs that propagate towards the cell soma. We hypothesized that when these APs reach the cell soma they would trigger opening of voltage-dependent calcium channels, resulting in a further increase in intracellular Ca^2+^ and producing a detectable signal (Figure 1A). Therefore, we stimulated the fluidically isolated axons with the appropriate agonist while monitoring the calcium response in the cell soma. Application of 100 nM capsaicin in the axonal compartment led to a quick activation of corresponding capsaicin-sensitive crossing (DiO-positive) neurons at 6 div and this response was represented as an increase in Ca^2+^ signal over time (Figure 1B). Similarly, direct depolarization of the axons with a 30 mM KCl solution was capable of producing Ca^2+^ responses at the soma (Figure 1A & B). We used two measures of axonal function in Ca^2+^ imaging experiments: the mean somal ∆F_ratio_ (see methods) and percentage of DiO-positive neurons responding to stimulation of axonal compartment. DRG axons showed a range of sensitivities to capsaicin, thus we determined the capsaicin dose response relationship in the axons using both measures. The average dose-response curves for capsaicin activation based on ∆F_ratio_ revealed an EC_50_ of 94 µM (log(EC_50_)= 1.97 ± 0.32, n = 3 cells per point) for axonal activation, while the relevant EC_50_ for somal activation was 135 µM (log(EC_50_)= 2.13 ± 0.34, n=3 cells per point, Figure 1C, left). We used somal stimulation with KCl as a confirmation of neuronal identity and cell viability. Comparison with DiO fluorescence confirmed that at 6 div the axons from the overwhelming majority of neurons cultured from neonatal rats have traversed through the microgrooves and into the opposite (axonal) side (Figures S1 and S2 in File S1). Therefore, all analysis was restricted to DiO-positive (crossing) neurons. In all experiments neurons that did not respond to KCl somal challenge were excluded from the analysis. More than 80% of analysed neurons on average could be axonally activated with 100nM capsaicin (93 ± 5%), while increasing the capsaicin concentration to 500 nM resulted in activation of 90 ± 6% of crossing neurons (Figure 1C, right). Finally, 72 ± 6% and 91 ± 6% of neurons responded to somal activation with low (100 nM) or high (500 nM) capsaicin, respectively. The EC_50_ values using percentage responders for axonal and somal stimulations were 19 (log(EC_50_) = 1.28 ± 0.16, n = 3 cells per point) and 47 µM (log(EC_50_)= 1.67 ± 0.13, n = 3 cells per point, respectively. These data confirm that imaging [Ca^2+^] in the soma is a robust and sensitive assay of axonal function in MFC cultures, using either ∆F_ratio_ or percentage of responders as functional readouts. 

**Figure 2:**
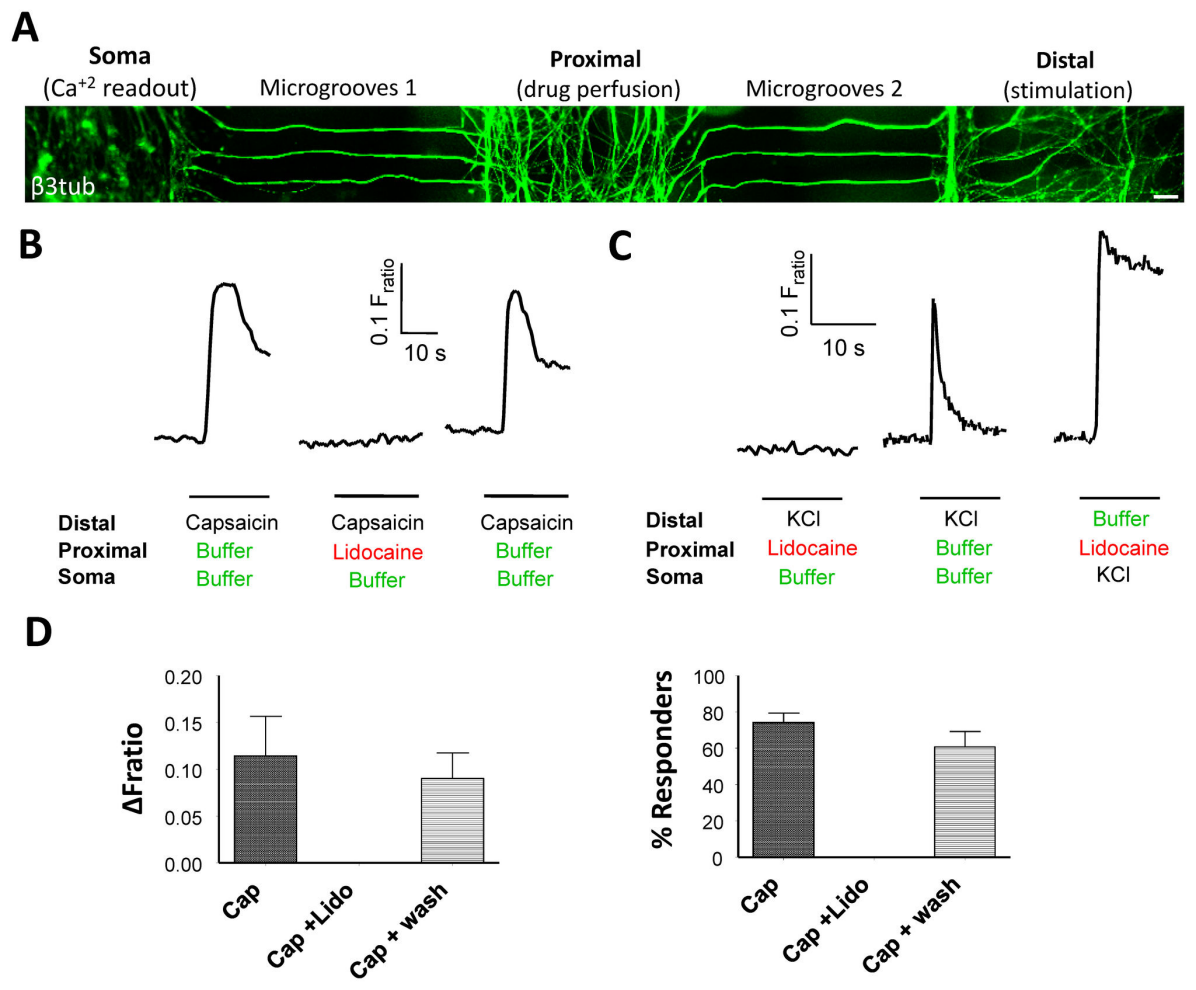
Using MFC cultures to selectively screen drug effects on axonal excitability and conduction. (A) DRG cultures in triple-compartment MFCs. Axons (stained here for β3tubulin) traverse two compartments growing through two sets of microgrooves; axons on the far right compartment (distal) can then be stimulated, while drugs are perfused through the middle compartment to study their effect on axonal conduction. Scale bar = 50 μm. (B) Left, representative calcium imaging traces illustrating axonal activation after capsaicin stimulation of axonal endings, which is completely blocked with concurrent perfusion of lidocaine (middle). After washing out the lidocaine, axons respond to a second stimulation (right). (C) Left, axonal responses to KCl stimulation are blocked by lidocaine in the proximal chamber. The presence of lidocaine in the axonal compartment does not inhibit KCl responses after somal application (right), illustrating the fluidic isolation property of microfluidic chambers. (D) Quantification of magnitude of Ca^2+^ response and percentage of responders to capsaicin with or without lidocaine (n = 3 independent cultures).

An important feature of the MFC system is the ability to screen the effects of a given drug on axonal excitability (AP generation) and signalling (AP conduction). We investigated modulation of axonal conduction by utilising a triple-compartment microfluidic chamber design consisting of somal, middle and axonal compartments and two sets of microgrooves inbetween. Neonate rat DRG neurons were seeded in the somal chamber and axons were allowed to grow through both sets of microgrooves. After 6 div, approximately 80% and 60% of neurons had crossed past the first and second set of microgrooves, respectively (Figure 2A). Like before, capsaicin stimulation at the axonal compartment resulted in a measurable calcium elevation in 75 ± 5% (n = 3 experiments, 5-10 cells each) of neuronal somata with an average ∆F_ratio_ of 0.115 ± 0.042 (n = 3 experiments, 5-10 cells each, Figure 2B, D and S3 in File S1). We then examined the effect of lidocaine, a topical anaesthetic that blocks membrane depolarization and AP generation/propagation through inhibition of fast voltage-gated sodium channels. As expected, the axonal capsaicin response in the distal compartment was completely blocked by perfusion of 10 mM lidocaine in the middle compartment. This effect was specific and reversible since drug washout restored the response in 60 ± 15% (n = 3 experiments, 5-10 cells each) of neurons with an average ∆F_ratio_ of 0.090 ± 0.027 (Figure 2D, left, and Figure S3 in File S1). Similar results were obtained when axons were stimulated with 30 mM KCl, with simultaneous lidocaine in either the middle or distal axonal compartments (Figure 2C). Importantly, inclusion of lidocaine in the axonal compartment did not prevent cell soma activation by KCl, demonstrating that there is no diffusion away from the fluidically isolated axonal compartment (Figure 2D, right). This result shows that the APs initiated in the axons propagated to the soma and are responsible for the Ca^2+^ influxes measured in the soma, hence illustrating the specificity of the MFC system. 

### Using MFC sensory neuron cultures as an *in vitro* model of nerve injury and axonal sensitization

**Figure 3:**
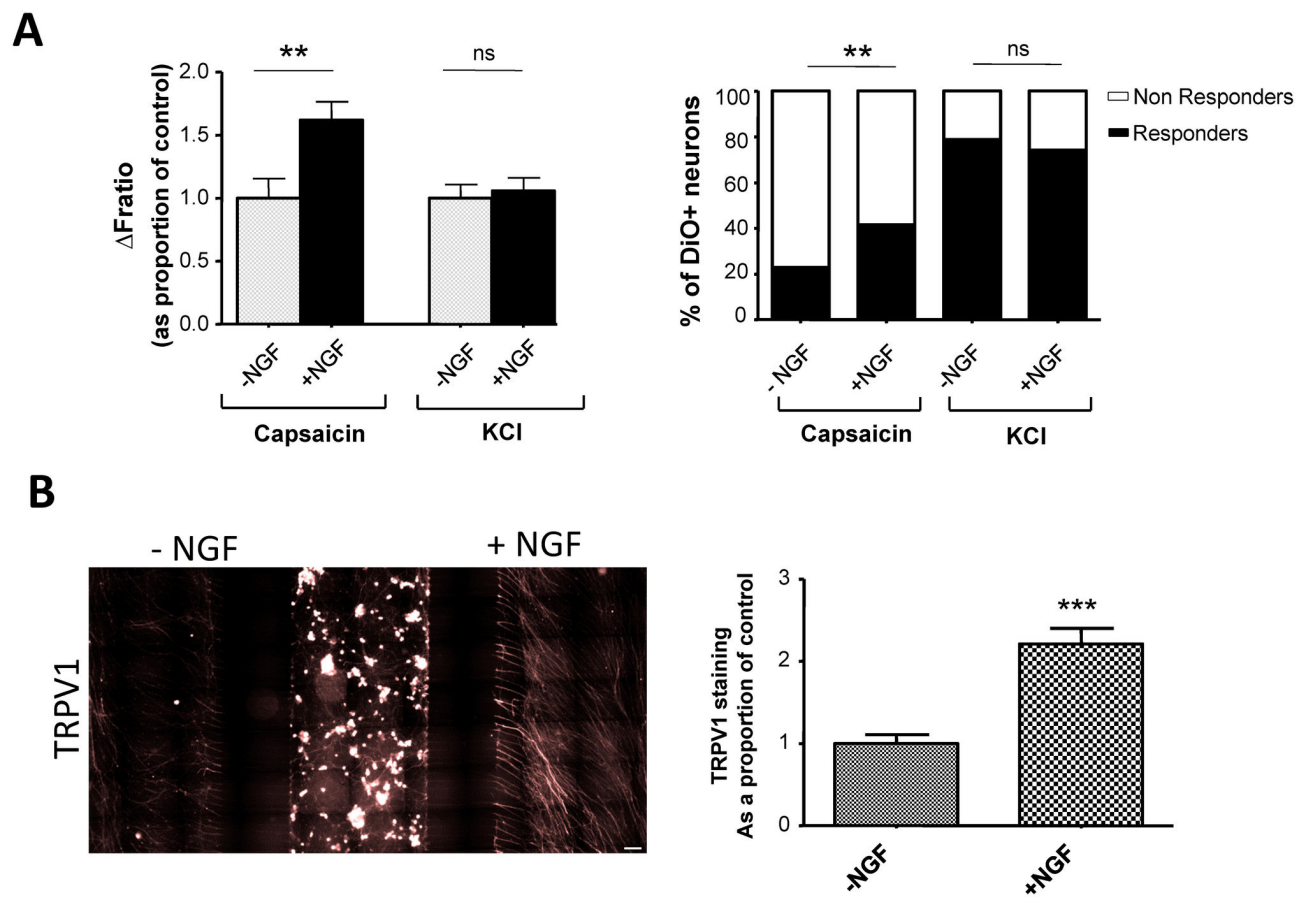
Local treatment of axons with NGF enhances capsaicin-evoked responses and TRPV1 expression in the axons. (A) Chronic NGF applied locally to axons induces a significant increase in capsaicin-induced excitability. Quantification of calcium imaging responses to axonal stimulation with capsaicin or KCl, in control cultures and cultures subjected to axonal NGF treatment. Left, magnitude of response (**p < 0.01, Fisher’s exact test, n = 70 and 89 from three independent experiments); right percentage of responders (**p < 0.01, one-way ANOVA with Bonferroni’s correction, n = 17 - 63 cells, three independent experiments,). (B) Axons treated with high NGF show increased local TRPV1 expression. Image is from a triple-compartment MFC in which neurons were seeded in middle channel and axons crossed on both sides. The axons on the left side were deprived of NGF while the right side was subjected to high NGF axonal treatment. Right, quantification of axonal TRPV1 immunoreactivity with or without NGF treatment (***p < 0.001, Student’s t-test, n = 47 and 37, three independent experiments). Scale bar = 100 μm.

DRG axonal dysfunction is a contributing factor in many of the chronic pain syndromes seen in patients (e.g. traumatic injuries, diabetic neuropathy, HIV-associated neuropathy). We therefore sought to develop *in vitro* axonal manipulations as a mimic for peripheral hypersensitivity and nerve injury. We initially investigated whether chronic (48 hrs) treatment of DRG axons with the pro-nociceptive NGF, a growth factor with an established role in inflammatory pain, can mediate peripheral sensitization changes [25]. To achieve this, neonate rat DRG cultures in MFCs were prepared as before, with the difference that an overnight NGF depravation step in both compartments was introduced at 3 div. Following this, the axonal compartment was treated with a high NGF concentration for 48 hrs, while control cultures received no further NGF. At 6 div, the average percentages of responders to axonal stimulation with capsaicin, but not KCl, were significantly increased by NGF treatment (capsaicin, from 22.9% to 41.6%, p = 0.017; KCl, from 91% to 96%, p > 0.05, Fisher’s exact test, n= 70 and 89 from three independent experiments; Figure 3A, and Figure S4 in File S1). Similar changes were observed when examining the intensity of Ca^2+^ signal (normalized ∆F_ratio_) in all responders (capsaicin, from 1.0 ± 0.15 to 1.62 ± 0.15, p < 0.05; KCl, from 1.00 ± 0.10 to 1.05 ± 0.1, p > 0.05; one-way ANOVA with Bonferroni’s correction, n = 17-63 cells, three independent experiments). The fact that no changes in axonal responses to KCl were observed, suggests that sensitization by NGF was not due to an increase in axonal length, arborization, or numbers of neurons crossing the microgrooves. Finally, we investigated whether NGF sensitization was coupled to changes in the expression of relevant axonal receptors. We used the triple-compartment configuration to examine regulation of TRPV1 expression by NGF, as this neurotrophin has previously been shown to increase TRPV1 function and trafficking [13]. For this, DRG neurons were seeded in the middle compartment leading to axonal crossing on both sides (Figure 3B). After overnight NGF deprivation as before, only one axonal side was treated with NGF according to the previous protocol. Quantification of total TRPV1 immunoreactivity revealed a 2.2-fold increase in TRPV1 expression in NGF-treated axons compared to untreated side (2.2 ± 0.2 to 1.0 ± 0.1, p < 0.001, Student’s t-test, n = 47 and 37, three independent experiments). Taken together, these results indicate that 48 hrs of axonal treatment with NGF increases the expression and function of TRPV1, leading to potentiation of local axonal responses independent of the soma.

**Figure 4:**
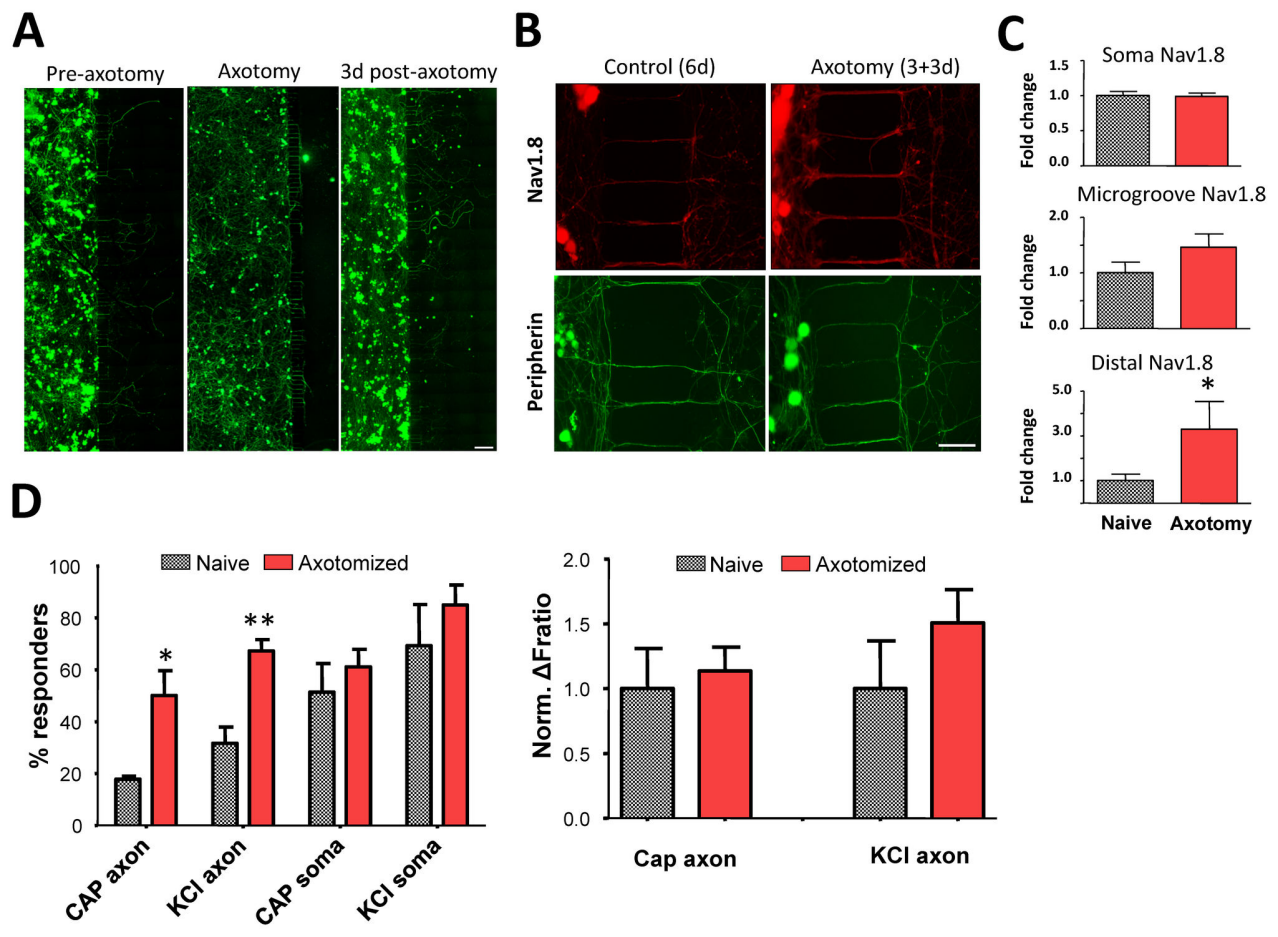
An *in* *vitro* model of traumatic nerve injury using adult mouse DRG cultures in MFCs. (A) Sensory neurons in MFC (left) can be axotomized *in* *vitro* using a suction pipette (middle), following which axons regenerate through the microgrooves (right). All images show staining for β3tubulin. (B-C) Axonal Nav1.8 expression is upregulated 3d after axotomy in the regenerating axons. Images show Nav1.8 staining. Right graphs show quantification of staining as fold change in Nav1.8 expression in the cell soma, microgroove-contained axons and nerve endings, before and after axotomy (*p < 0.05, Student’s t-test, n = 3). (D) Axotomized neurons are sensitized compared to control neurons, evidenced by the increased percentage of responders after axonal stimulation with capsaicin or KCl (left, *p < 0.05, **p < 0.01 vs naïve, Student’s t-test, n = 3 independent experiments). The magnitude of Ca^2+^ responses to axonal stimulation following axotomy was not significantly different compared to control neurons (right). Scale bars: A = 200 μm, B = 50 μm.

We next assessed how direct axonal damage in MFC affects axonal function. We used DRG neurons from adult mice which show robust development of hypersensitivity in animal models of nerve injury [26]. MFC cultures were grown according to standard protocols for 3 div, at which point we induced *in vitro* axotomy of crossing axons by applying suction through a fine glass pipette positioned at the entrance of the axonal compartment (Figure 4A). Axotomized MFCs were left for an additional 3 div, during which damaged axons were able to regenerate back into the axonal compartment. At 6 div, the total axonal growth in the axonal compartment was approximately 57.1 ± 0.1% of matched control cultures (6 div with no axotomy), as quantified by peripherin immunoreactivity. Interestingly, using calcium imaging we observed a significant increase in the percentage of neurons in axotomized MFC cultures responding to axonal stimulation by capsaicin or KCl, compared to naive (Figure 4D and Figure S5 in File S1; capsaicin, 17.8 ± 1.1% vs 50.0 ± 9.6%; KCl, 31.7 ± 6.3% vs 67.2 ± 4.3%, Student’s t-test, p < 0.05 and p < 0.01 respectively, n = 3 for each). 

We asked whether the increased axonal responses after axotomy are due to expressional changes in sodium channels. To answer this question we performed immunocytochemistry for Nav1.8, since this sodium channel has a pivotal role in AP generation and inflammation-induced hyperexcitability [27]. Quantification of Nav1.8 immunoreactivity in axotomized and control cultures indicated that there was no difference in Nav1.8 levels in the cell soma or axonal surface in the microgrooves (Figure 4B, C). Intriguingly however, Nav1.8 signal in the ‘regrown’ segments of axotomized axons showed a 3.3-fold increase compared to control (Student’s t-test, p = 0.01, n = 3). This finding suggests that *in vitro* axotomy of Nav1.8-positive neurons leads to increased channel expression in the regenerating portion of damaged axons, similarly to what has been reported in painful neuromas [28,29]. 

In summary, our data illustrate that axonal sensitization in MFC can be used as an *in vitro* model that recapitulates important aspects of *in vivo* peripheral hyperexcitability following inflammation or nerve lesion.

### Using patch-clamping to assay electrophysiological characteristics of axons in the MFCs

**Figure 5:**
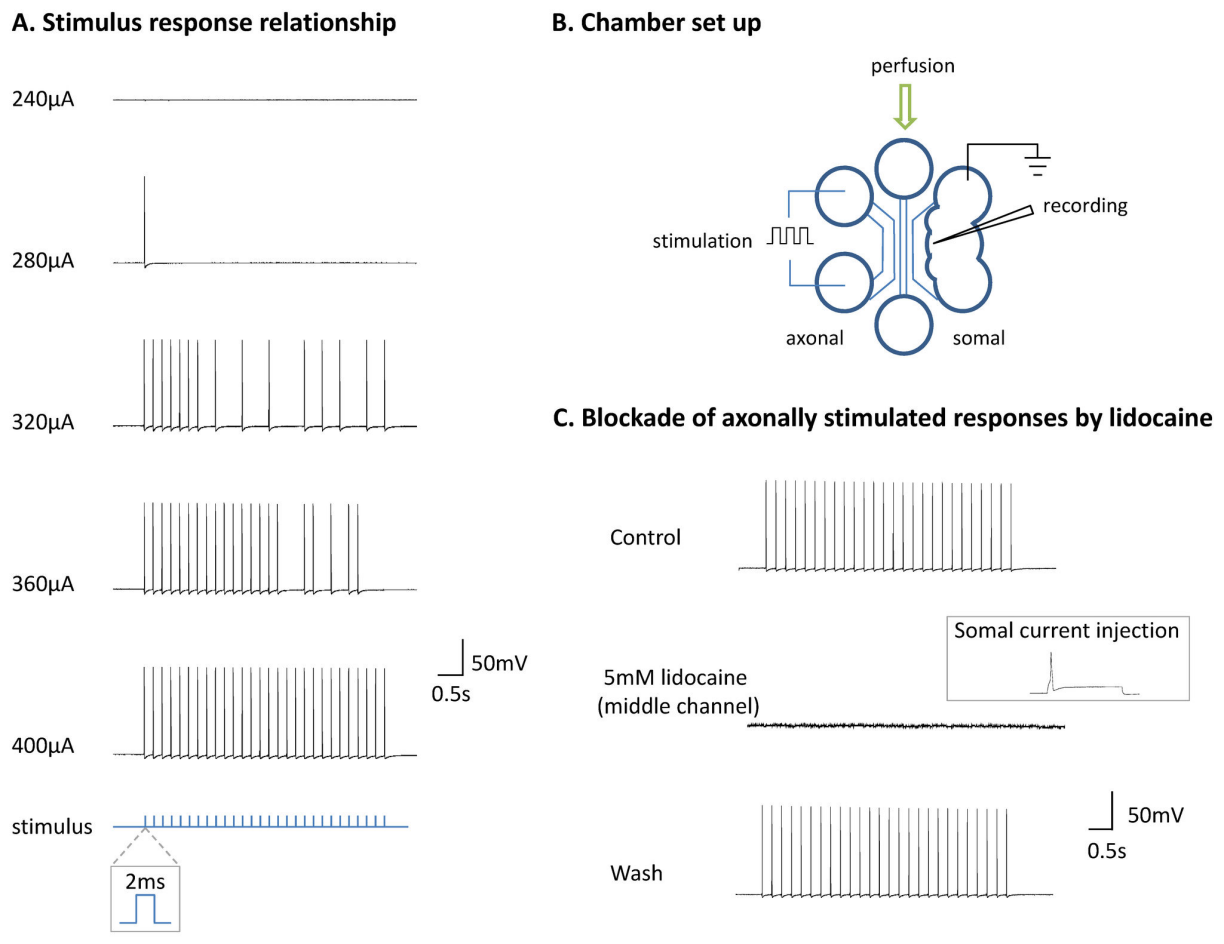
Electrical stimulation to generate axonal APs in the MFC. (A) Electrical stimuli applied to the axonal chamber consisted of trains of constant current stimuli of 2 ms duration applied at 5.6 Hz for 5 s, with 20 s inter-train intervals. Stimuli produced somal APs which increased in fidelity as the stimulus intensity was increased. Stimulus intensities for each train are shown on the left. (B) The configuration of the triple-compartment electrophysiological setup. Whole-cell current clamp recordings are made from cell bodies in the somal compartment. Perfusion is applied through the middle axonal channel with electrical stimulation applied in the far axonal channel. (C) Application of lidocaine blocks axonally stimulated responses. Control responses are shown (top trace), where each stimulus resulted in a somal AP. Application of 5 mM lidocaine in the middle axonal channel abolished somal spikes, whereby somal stimulation could still elicit an AP (see inset). Washout reversed the inhibitory effects of lidocaine. All stimuli were suprathreshold at 1.2 mA.

We have shown how to use MFCs to independently alter properties of the axons/terminals either by pharmacological or by physical lesions (*in vitro* axotomy) and consequently assess the functional impact of these changes on somal responses with Ca^2+^ imaging. To study generation and propagation of APs in axons we modified commercially available MFCs for electrophysiological recordings (Figure 5B). Holes were punched in the chambers on one side to allow DRG neurons to be plated near the microgrooves yet remain accessible to micropipettes in order to carry out patch-clamp electrophysiology on the cell soma. The 6-well microfluidic configuration permitted the use of separate channels for electrical stimulation of axons via silver wire electrodes (far axonal channel) and perfusion of pharmacological agents (middle axonal channel). 

Application of electrical stimuli in the far axonal channel produced APs in the axons that reached the cell soma. Example recordings of this are shown in Figure 5A. Periodic trains of stimuli were used to help prevent significant run down of the responses and thresholds for firing with the constant current stimulator ranged from 38 to 480 μA with stimuli of 2 ms duration. Initially, cells could not faithfully follow the stimulus train, however with increasing stimulus intensity the fidelity of the response increased such that cells eventually responded with an AP to every train stimulus (Figure 5A, bottom trace). 

To test whether the applied electrical stimulus was reaching the somal compartment, and thus directly stimulating the cells, we applied suprathreshold stimuli in the far axonal compartment whilst blocking axonal activity in the middle channel with a high concentration of lidocaine (Figure 5C). Lidocaine successfully blocked axonally stimulated responses in the soma, suggesting that the stimulus does produce an axonal AP which travels to the soma. Additionally, when the soma was stimulated directly with current injection in the presence of lidocaine in the middle axonal compartment, APs could still be produced (Figure 5C, inset), indicating that the soma is still viable and that blockade only affects conduction in the axon. This result also confirms that fluidic isolation of the compartments is maintained during drug perfusion. 

### Axonal application of sodium channel blockers alters electrophysiological parameters of AP transmission to the soma

**Figure 6:**
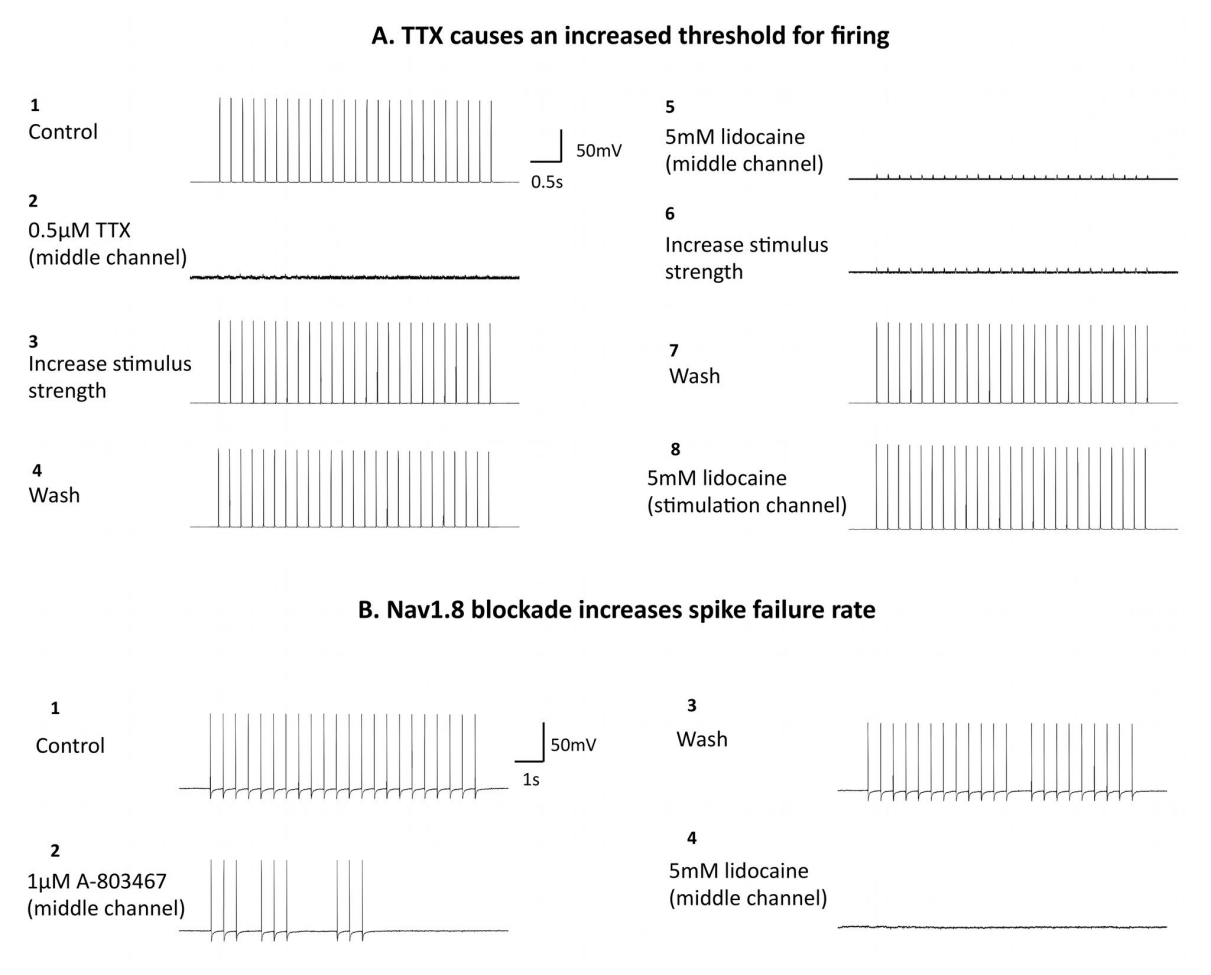
Using patch clamp electrophysiology to study impact of sodium channel blockers on axonal function. (A) The top trace shows control somal responses to electrical stimulation of axons (1 mA, 2 ms duration stimuli, applied in 5 s, 5 Hz trains, with 20 s inter-train intervals). Addition of 0.5 μM TTX to the middle axonal channel completely inhibited somal APs, however increasing stimulus intensity to 2 mA restored the somal response. Subsequent application of lidocaine to the middle channel in this cell abolished responses which could not be restored with higher stimulus intensities. Washout reversed the inhibitory effects of lidocaine. Application of lidocaine to the far stimulation channel (bottom trace) had no effect on the somal response. (B) The top trace shows control responses to electrical stimulation of axons (5 mA, 2 ms duration stimuli, applied in 10 s, 2.2 Hz trains, with 10 s inter-train intervals). The addition of the Nav1.8 blocker A-803467 (1 μM) to the middle axonal channel in this cell increased the somal spike failure rate. Washout reversed the effects of A-803467 and the lower trace shows complete abolition of somal responses following axonal application of lidocaine (middle channel).

One important application of this technique is the ability to selectively manipulate the axonal membrane using pharmacological tools to examine the involvement of various ion channels in transmission of impulses to the cell soma. We tested two blockers of voltage gated sodium channels – the primary ion channels responsible for axonal impulse transmission (Figure 6). In the 6-well configuration, application of 0.5 μM tetrodotoxin (TTX) in the middle axonal channel appeared to abolish the response to electrical stimulation (Figure 6A2). However, doubling the stimulus intensity restored the response suggesting that the threshold for firing had been increased by the specific blockade of TTX-sensitive sodium channels in the axon (Figure 6A3). Following a wash period, a high concentration of lidocaine was added to the middle axonal channel and, as shown previously, was able to abolish responses suggesting that the electrical stimulation was limited to the axonal compartment (Figure 6A5). In this instance increasing the stimulus strength up to the maximal intensity could not restore responses (Figure 6A6), suggesting that lidocaine has a more potent inhibitory effect than TTX, likely due to its less subtype-specific voltage-gated sodium channel block.

Lidocaine was also applied to the stimulation channel (Figure 6A8) but was found to have no effect on axonally stimulated somal responses. This suggests that the initiation point of the AP in response to electrical stimulation in these cells is located somewhere between the far and middle axonal chambers (as lidocaine blocks APs in the middle channel). We also examined the effect of the more specific sodium channel blocker A-803467, a selective inhibitor of the TTX-resistant channel Na_V_1.8 which has been shown to change the firing properties of nociceptive axons *in vivo* [30]. Application of 1 μM A-803467 in the middle axonal channel caused an increase in the spike failure rate whereby the cell could no longer produce a response to each stimulus in the train, suggesting that axonal Na_V_1.8 sodium channels are required for faithful conduction of impulses to the soma (Figure 6B2). This effect was reversible upon washout and subsequent blockade of axonally driven responses by lidocaine in the middle channel again confirmed the stimulus was not crossing to the somal side (Figure 6B3-4). 

Taken together, these results show that pharmacology can be successfully used in the MFC electrophysiology setup as a tool to investigate the functional roles of axonal ion channels in AP transmission to the soma.

### Using MFCs to examine electrophysiological changes in injured axons following axotomy

**Figure 7:**
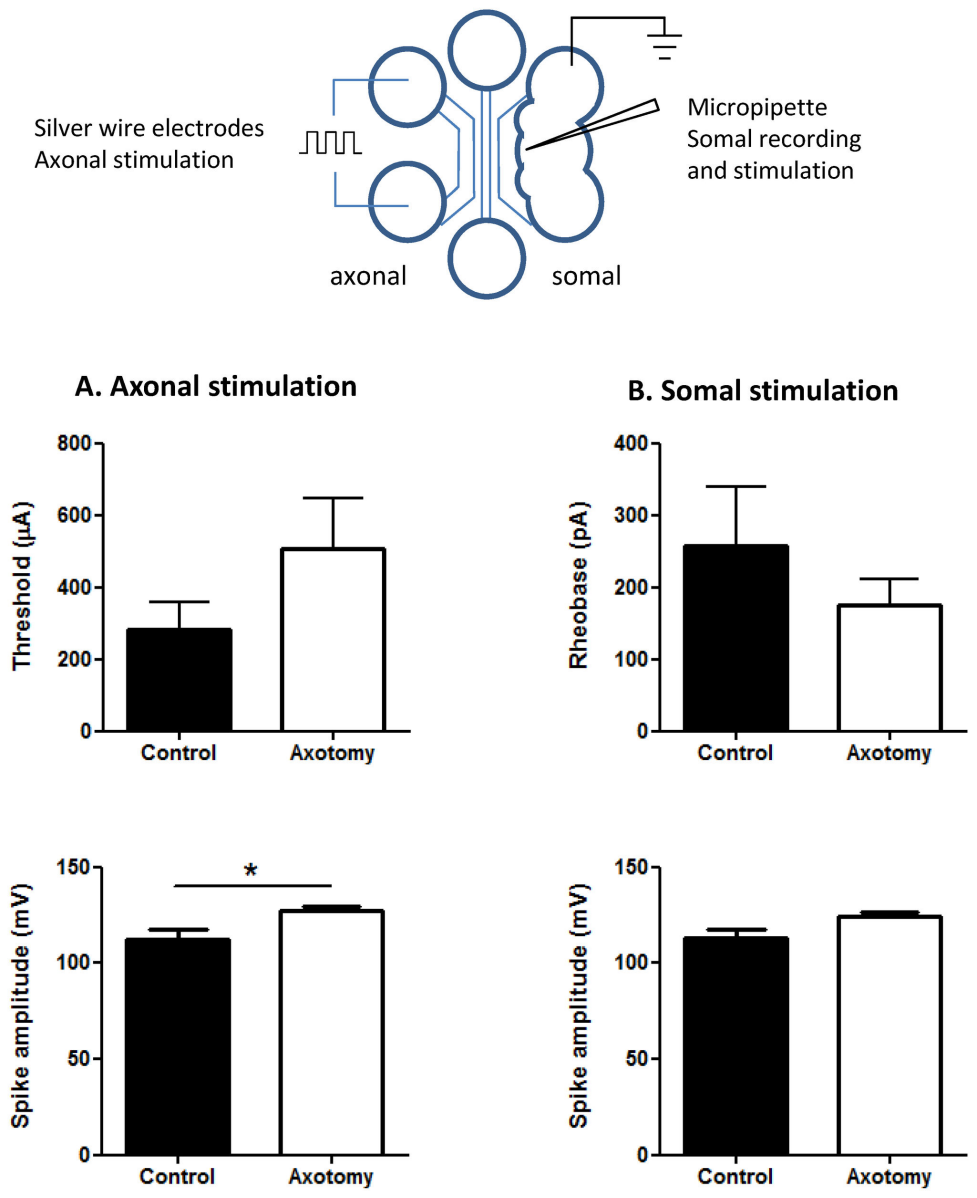
MFCs can be used to examine the electrophysiological effects of *in* *vitro* axotomy. (A) Following *in* *vitro* axotomy there was no significant difference in the somal AP threshold to axonal stimulation, however a new population of cells with higher thresholds appeared (upper panel). Axotomy produced a significant increase in the axonally stimulated somal spike amplitude (*p < 0.05, lower panel). (B) In vitro axotomy caused no significant difference in the somal AP rheobase or spike amplitude following direct somal stimulation (control, n = 6; axotomy, n = 5 for all data).

We have shown an increase in proportion of responding neurons in axotomized MFCs and a parallel increase in Nav1.8 staining in regenerating axons. We then aimed to test whether this axotomy-induced change results in augmentation of individual axonal activation. We examined electrophysiological parameters of DRG neurons following *in vitro* axotomy as described previously. Three days following axotomy there was a significant increase in the AP size in response to axonal stimulation (Figure 7A, lower panel, p < 0.05, n = 8 and 9, Student’s t-test). In addition, a sub-population of cells with increased axonal activation thresholds was observed, although this did not lead to an overall significant increase in the average threshold value (Figure 7A, upper panel). Somatic stimulation of axotomized neurons revealed no significant difference between control and axotomized cells in either spike amplitude or rheobase for firing, although there was a trend for the former to increase, and the latter to decrease (Figure 7B). Hence the observed increase in AP amplitude in axonal stimulation of axotomized MFCs is consistent with the increase in Nav1.8 expression we observed in regenerating axons. These results highlight the potential of this technique for studying changes in the biophysical properties of axons following *in vitro* axotomy. 

### Application of MFC to study axonal interactions with other cell types

**Figure 8:**
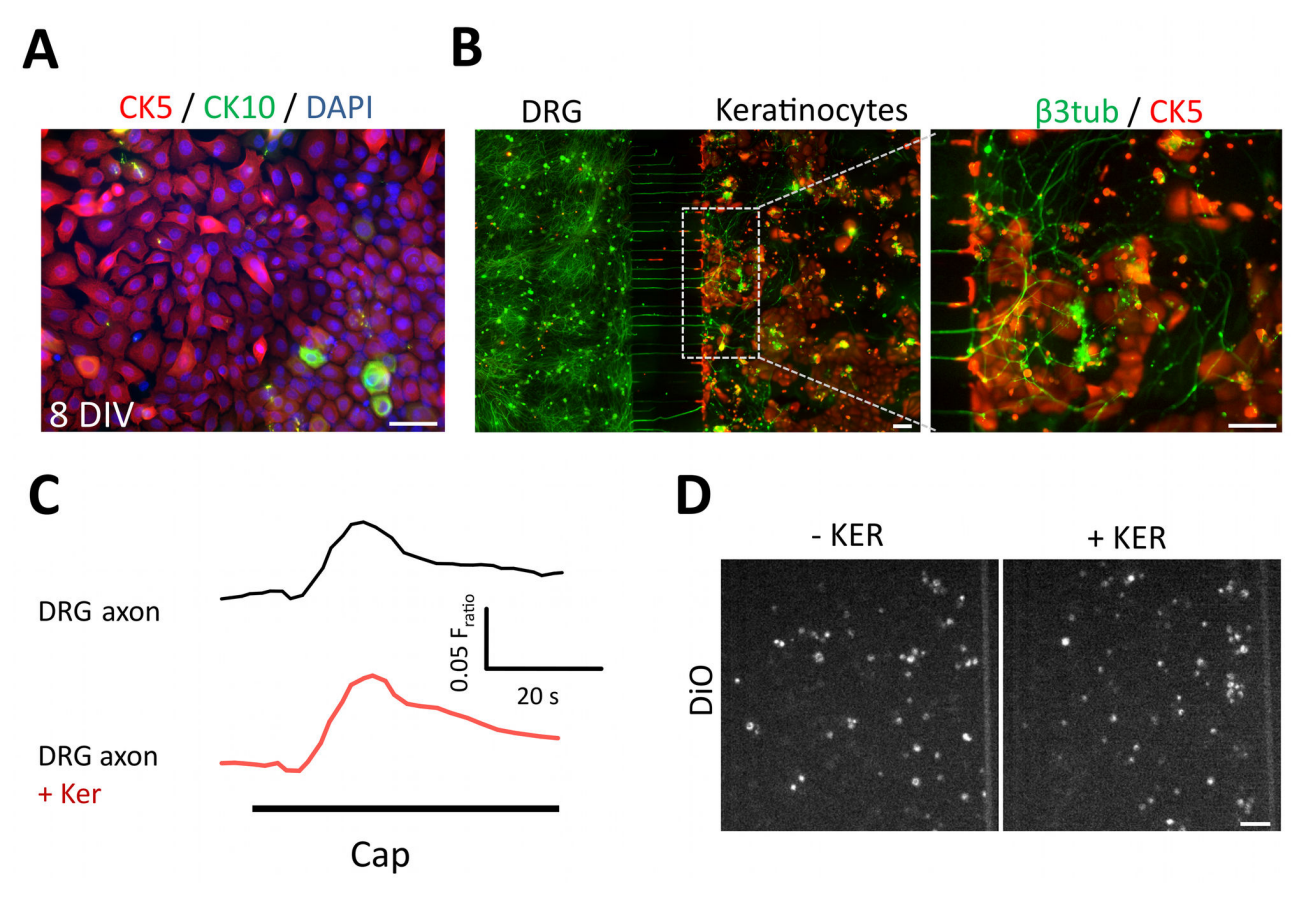
Using MFCs to study axonal function in DRG keratinocyte co-cultures. (A) Rat neonatal keratinocytes culture in MFC. Immunocytochemistry for basal keratinocyte marker cytokeratin 5 and differentiation marker cytokeratin 10, illustrating low levels of differentiation after 8 div in MFC. (B) A co-culture of rat neonate sensory neurons (stained for β3tubulin, left compartment) and keratinocytes (stained for cytokeratin5, right compartment) in MFC at 6 div. (C) Example traces of axonal activation in the presence or absence of co-cultured keratinocytes in the axonal compartment, as measured by Ca^2+^ imaging in response to capsaicin stimulation of axons. Scale bars = 100 μm. (D) Co-cultured keratinocytes did not impede crossing of DRG axons. There was no difference in tracer (DiO) uptake between cultures grown with or without keratinocytes, indicating similar levels of axonal crossing.

A particularly attractive extension of the MFC platform is the ability to study the effect of neuronal (e.g. sympathetic, dorsal horn neurons) or non-neuronal (e.g. immune cells) cell types on axonal excitability. In a proof-of-principle experiment, we utilised the MFC system to co-culture rat sensory neurons with keratinocytes which are in contact with nociceptive endings in the skin and increasingly reported to play a role in nociceptive modulation. In this preparation, DRG neurons were plated in the somal compartment while keratinocytes seeded in the axonal compartment, each with their own growth medium. Because keratinocyte proliferation in culture can lead to differentiation and programmed cell death (terminally differentiated epidermal keratinocytes), we first characterised keratinocyte growth. Under the MFC culture conditions the vast majority of keratinocytes remained undifferentiated for up to 8 div, as revealed by the expression of basal keratinocyte marker cytokeratin-5 (Figure 8A). In contrast, very few cells were stained positive for the marker cytokeratin-10, which stains differentiated cells [31]. In these co-cultures, neurons extended axons through the microgrooves and occupied the space above and in between keratinocyte patches, where extensive branching was observed (Figure 8B). No effect of keratinocytes on neuronal viability or percentage of crossing neurons was observed (Figure 8C). Finally, we used calcium imaging to examine the intensity of axonal activation in the co-presence of keratinocytes (Figure 8D). The results suggested that co-culturing of DRG with keratinocytes did not alter basal axonal excitability in response to capsaicin or KCl stimulation, compared to matched cultures with DRG neurons alone. Therefore, this microfluidic-based co-culture system provides the ability to investigate the role of interacting cells (e.g. keratinocytes) in modulating axonal function.

## Discussion

Understanding local responses in axons and the signalling pathways that orchestrate these responses requires the ability to study the axonal compartment in isolation from the rest of the cell. Although devices such as Teflon-based Campenot chambers have been successfully integrated in neurobiology their inherent limitations, namely crosstalk between chambers, manufacturing or assembly complexities and lack of compatibility with sophisticated imaging techniques, restricts their usage [32,33]. However, the development of microfluidics methodology has greatly extended the application of compartmentalized cell culture platforms in neurobiological studies [6]. Microfluidics technology is increasingly applied to diverse neurobiological questions such as developmental regulation [34,35], formation of synapses and functional connectivity [34,36], regeneration [22,37], axonal transport and local protein synthesis [18,38,39]. Here, we have expanded the microfluidics platform by developing a user-friendly, powerful and versatile system to study *in vitro* responses of DRG axons as a surrogate model of free nociceptive nerve endings. We show that, firstly, isolated axons belong to sensory neurons that express classical nociceptive markers. Secondly, these nociceptors can be axonally stimulated by algogens while calcium imaging or patch-clamp recording in the cell body provides a read-out of the axonal activation. Thirdly, experimental manipulations localized to axons can bring the system to a sensitized state reminiscent of the peripheral sensitization encountered in chronic pain. Fourthly, the MFC platform allows axonally restricted co-cultures to investigate modulation by other cell types such as keratinocytes. 

Mature DRG neurons constitute a heterogeneous population of cells distinguished on the basis of protein expression, neurotrophic factor dependence, conveyed sensory modalities and innervation targets and the MFC cultures reflect this diversity. We show that the isolated axons and associated sensory neurons are predominantly nociceptive and express typical phenotypic markers (CGRP and IB4), ionic machinery membrane proteins (sodium channels) and pain receptors (TRPV1), all characteristic of mature excitable pain-sensing neurons [8]. Examining the distribution of crossing cells in MFC, we observed a bias towards smaller CGRP and IB4 -positive cells (axons), which is similar to conventional DRG cultures. A smaller number of NF200-positive axons were detected in the axonal compartment, which may be indicative of medium-large neuronal subpopulations that are reported to express GFRα-1 receptor components and are therefore regulated by GDNF [40]. 

We sought to develop an *in vitro* platform to study axonal generation and propagation of APs independently of the soma, hence recapitulate the way impulses are generated and propagated *in vivo* in a controlled and accessible environment. We showed that in our system stimulation of axons either chemically (capsaicin or KCl) or by electrical pulses, results in generation of APs that propagate down the axons and depolarize the soma on arrival, resulting in elevated Ca^2+^ concentrations. Comparison of the changes in somal Ca^2+^ concentrations in response to axonal versus somal stimulation showed that for the majority of axons, stimulation results in a smaller calcium signal, reflecting the different pathway that leads to elevated somal Ca^2+^ in each case (i.e. depolarization by propagating APs versus direct depolarization of somal membrane). Hence the system is particularly suited for rapid screening of compounds to ascertain the role of their targets in axonal excitability. In our proof-of-principle study, axonal application of lidocaine, a topical anesthetic blocker of Nav channels [41], completely abolished AP generation and propagation along the axon, while somal stimulation responses remained unaffected. These results confirmed that in our system the fluidic isolation previously documented with other methods [21] remains intact during axonal stimulation and demonstrates the utility of the system in investigating the function of local ion channels or second messenger pathways.

A number of methodologies have been used to study characteristics of nerve conduction and nociceptive responses, including the skin-nerve and isolated ganglia nerve preparations [4,5], however these techniques require specialized instrumentation and expertise and are not readily suitable for patch-clamp recording. We show that with minimal modification, the MFC system becomes compatible with patch-clamp configuration to carry out detailed analysis of the electrophysiological characteristics of axons. As proof of principle, we assessed axonal responses to repetitive electrical stimulation and showed that TTX-sensitive channels (primarily Nav1.7 [42]) are likely involved in setting thresholds of activation in axons, whereas analgesic compounds such as A-803467 that selectively block Nav1.8 channels result in increased conduction failure. Interestingly, a reduction in conduction failure has been suggested to contribute to neuropathic pain conditions such as painful diabetic neuropathy [43]. The current configuration of MFC used in this study does not allow separation of the effect on initiation and conduction of APs, since the exact location where electrical stimulation results in generation of APs cannot be definitively determined. Based on our observations however, we believe that AP initiation occurs at a midpoint along the axons traversing the microgrooves or slightly into the middle chamber. Refinements in the design of the MFC, such as extending the microgroove length, could be used to enhance localization of the electrical stimuli. 

Chronic pain conditions are accompanied by significant peripheral hyperexcitability of nerve fibers [44]. We looked at axonally-restricted manipulations such as NGF treatment or physical lesion (axotomy) *in vitro* and observed axonal hyperexcitability within 72 hrs, consistent with observations in inflammatory and neuropathic animal pain models [45–47]. NGF-mediated hyperexcitability has been of great interest for its therapeutic potential. We show that axonal NGF treatment leads to enhanced capsaicin responses and demonstrate that this is most likely due to local increase in axonal TRPV1 expression, in agreement with previous reports [13,48]. Hence local axonal pathways activated by NGF are highly relevant to the associated hypersensitivity in inflammatory pain. 

Pain caused by nerve transection has been linked to accumulation of sodium channel subunits in proximal stumps and neuromas [29]. Interestingly, we observed an increase in Nav1.8 imuunoreactivity following *in vitro* axotomy in the regenerating axons, which may account for the increased axonal excitability we detected. It would be interesting to ascertain whether specific Nav1.8 inhibition can restore neuronal excitability in the re-growing axons. However, additional channels and mechanisms may also contribute to the post-axotomy phenotype [49–52]. In addition, a substantial body of literature has documented the effect of priming DRG injuries in shaping subsequent nociceptive pathophysiology and pain behaviours [53,54]. Therefore, a future refinement of the MFC system is to assess axonal responses following *in vivo* priming such as exposure to proinflammatory mediators [55]. Alternatively, DRG neurons from neuropathic animals could be cultured to examine how *in vivo* priming lesions compare to *in vitro* axotomy, and whether these manipulations lead to cumulative changes in axonal excitability [56]. Another intriguing possibility is to use the L5 spinal nerve transection model to compare axonal properties of axotomised L5 versus spared L4 DRG neurons [57]. In summary, axotomized neurons in MFC cultures constitute a relevant model of *in vivo* nerve lesions applicable to diverse experimental scenarios.

An important aspect of DRG axonal physiology is the interactions with non-neuronal cells that often serve to regulate the responses to injury. Although mixed cultures of DRG neurons with other cell types have been developed, they lack the finely regulated layered organization present in whole organisms [58]. For instance, axons innervating the skin are subject to modulation by the target microenvironment, and keratinocytes can induce axonal branching and CGRP upregulation in small DRG neurons [59]. In our paradigm, we used MFC to characterize basal axonal responses in the presence of keratinocytes, as emerging data suggests an active role of these cells in pain processing [60]. It would be interesting to decipher whether keratinocytes further augment axotomy-induced axonal hyperexcitability in MFC as reported for *in vivo* skin grafts [61]. It should also be noted that in order to establish a more physiological representation of the native DRG microenvironment, neurons were grown in the somal chamber together with their co-isolated supporting cells. This did not pose a problem for the specificity of axonal characterisation, as the microgroove barrier precluded the presence of these cells in the axonal compartment. Nevertheless, we have successfully applied mitotic inhibitors (e.g. cytosine arabinoside, not shown) to limit non-neuronal cell growth in the somal compartment, an implementation that could be used to probe the role of supporting cells in neuronal excitability.

Several additional avenues relevant to pain research could be pursued using the MFC system. For instance, chemotherapy-associated conditions such as vincristine and oxaliplatin-induced neuropathies have been attributed to cytoskeleton disruption that leads to defects in axonal transport [62]. Using MFC, it would be relatively straightforward to test whether axonal oxaliplatin treatment produces similar sensitization effects to somal treatment [63] and whether this phenotype can be reversed by inhibition of axonal translation or transport [64]. Analogous principles could be applied to study axonal degeneration in peripheral ‘die-back’ neuropathies such as diabetic-neuropathy [65]. 

In summary, we have described a novel MFC based platform for *in vitro* study of axonal function. We show the utility of the system to model axonal injury, sensitization and interaction with non-neuronal cell types. The response properties, changes in excitability and other electrophysiological parameters of axons can be conveniently probed using Ca^2+^ imaging or patch-clamp recording at the cell soma. We provide data on nociceptive axons and pain signalling, however the platform can readily be extended to study axonal function of other neurons in the context of diverse neurological disorders. 

## Supporting Information

File S1 **Supplementary Information**.
Figure S1, Cultures of neonatal and adult DRG neurons thrive in MFCs. Figure S2, Phenotypic characterization of DRG cultures in MFCs. Figure S3, Example of axonal stimulation evoked responses in somal compartment blocked by lidocaine in a 6 well MFC configuration. Figure S4, Example axonal capsaicin responses in two MFCs after 48 hr in which the axons were treated with either NGF or anti-NGF antibodies. Figure S5, Example of responses to stimulation of axotomized mouse DRG axons in MFCs 72 hrs post axotomy. Methods S1. Results S1.
(DOCX)
