# Supplementary material for: Probing Functional Properties of Nociceptive Axons Using a Microfluidic Culture System
Source: PLoS One. 2013 Nov 20;8(11):e80722. doi: 10.1371/journal.pone.0080722 (PMC3835735; doi:10.1371/journal.pone.0080722)

**Supplementary Results**

**Preparation and maintenance of sensory neurons in microfluidic cultures**

We first sought to create and optimize a microfluidics-based culture system that can be used to study axonal excitability *in vitro*, using dissociated DRG neurons isolated from neonate rats or adult mice. Sensory neurons were seeded in the somal compartment of the assembled device and cell growth was monitored over days in culture (Fig. S1). Axonal processes were visible within a few hrs in culture and by 12 hrs axons had entered the microgrooves. By 24 hrs post-plating the first axons had crossed over to the axonal compartment, while by 6 days *in vitro* (div) multiple axonal crossings typically through all microgrooves were visible. In cultures from postnatal neurons a few non-neuronal cells would be visible in the axonal compartment after 6 div, however this was not observed in the adult DRG cultures. Axonal crossings were presumably due to neurons seeking out the high neurotrophin concentration in the axonal compartment, although hydrostatic pressure towards the axonal side is likely to also play a crucial role. The percentage of neurons crossing could be monitored in real time via inclusion of a fluorescent tracer (DiO) in the axonal compartment (Fig. S1B). The tracer was taken up by axons and retrogradely transported back to the cell soma where the fluorescent signal accumulated to detectable levels within a few hours. Importantly, non-specific diffusion of the dye back to the soma compartment was prevented by the MFC fluidic isolation. Thus, at 3 div several (but not all) neurons were positive for green fluorescence as determined by live imaging. Neurons closer to the microgrooves tended to feature stronger signal, presumably due to increased crossing probability in the first hours of culture. At 6 div a higher proportion of neurons were positive and the signal intensity was more uniform across all cells. The percentage of crossing neurons at this time point was typically > 60%, with some preparations exhibiting crossing as high as 80%. There was no further increase in axonal crossings with more days in culture thereafter. The majority of neurons were small size, although a minority of large neurons could also be identified. No apparent toxicity or excessive cell death due to the PDMS material or culture conditions was observed at any point, and MFC could be retained for at least 14 days. Finally, we successfully applied the system to grow DRG cultures from adult mice (Fig. S1C). Because adult axonal growth appeared slower compared to neonate rats, we decreased the length of the microgrooves by a third (down to MFCs with 150μm-long grooves) and found that at 6 div in these optimized conditions the axonal crossings were comparable to neonates.

**Phenotypic characterization of sensory neurons in MFC**

Subpopulations of sensory neurons express distinct markers, receptors and ion channels and are differentially regulated by trophic factors. These variant properties eventually translate into differential involvement in aspects of nociceptive signaling. Therefore, we carried out expressional profiling using immunocytochemistry to neurochemically characterize the cell soma and axons of crossing neurons from neonate rats at 6 div.

In order to ascertain whether cultured neurons and associated axons are phenotypically similar to *in vivo* DRG neurons capable of conducting APs, we examined the expression of sodium channels using a pan-sodium channel antibody (Fig. S2A). Strong staining was observed in the cell soma of the vast majority of neurons contained in the somal compartment. Importantly, sodium channel expression was evident along axonal segments contained in the microgrooves, as well as axonal endings in the axonal compartment. In the latter, sodium channel localization appeared more punctate and structures indicative of growth cones could also be distinguished.

An important classification of sensory neurons is in small nociceptors, which can be further subdivided into peptidergic and non-peptidergic, and large neurons which are predominantly low threshold mechanoreceptors. By investigating co-localisation of neuronal markers with the fluorescent tracer it was possible to decipher the neurochemical identity of crossing cells (Fig. S2B and S2C). Thus, 36.3 ± 1.2 % of crossing neurons expressed the peptidergic marker calcitonin gene-related peptide (CGRP), while 32.3 ± 5.0 % of neurons were found positive for the non-peptidergic marker isolectin B4 (IB4). Interestingly, a minority of large neurons (17.0 ± 3.3 %) were immunoreactive for neurofilament-200 (NF200) (n = 3 independent cultures, mean ± SEM). In summary, the phenotypic characterization data demonstrate that all typical sensory neuron subpopulations are represented in the MFC system.

**Supplementary Methods**

**Preparation of microfluidic chamber (MFC) cultures**

Pre-sterilized 40mm-diameter glass bottom dishes (WillCo Wells) were coated with 0.5mg/ml Poly-L-Lysine at 37 °C overnight. The following day dishes were rinsed three times with water and subsequently coated with 20μg/ml laminin for 2 hours at 37 °C. Microfluidic devices (Xona Microfluidics) were sterilized in 70% ethanol for 5 min and left to dry out for at least 30 min. The SND450 design (microgroove length of 450μm) was chosen for neonate rat MFC, while for adult mouse cultures the SND150 option was used (150μm microgrooves). In the experiments utilising the triple compartment design, we used the TCND500 model (two 500 μm microgroove barriers with a 500 μm central chamber). For attachment of the device, the microfluidic chamber was placed on the coated glass bottom with the imprinted (microgroove) side down and gentle but firm pressure was applied with forceps to form a seal. An equilibration step followed, for which 200μl and 100μl of HBSS were added to the top left (somal compartment) and top right (axonal compartment) wells, respectively. The liquid was then forced through the channel towards the interconnecting well by forcefully pipetting at the channel entrance, taking care to avoid bubble formation. Finally, the chamber was left to equilibrate for 1 hour at RT to establish liquid flow through the microgrooves. For cell loading, liquid was removed from all wells as well as somal channel using a suction glass pipette and 5μl of cell suspension containing 4-6 x 10^5^ cells were added at the entrance of the somal channel. Cells would typically enter the channel and move slowly towards the bottom well of the somal compartment. When movement was restricted due to high cell density or debris presence, gentle suction was applied to the channel exit to assist homogenous cell dispersion. Finally, MFC were placed in the incubator at 37 °C for 1hr to allow cell attachment, following which more media was added; each well of the somal compartment was supplemented with 200μl Neurobasal, while the axonal compartment wells were filled with 100μl media each. In triple compartment cultures, the middle compartment wells were supplemented with 150 μl of medium. During this step, extra care was taken to minimize liquid flow through somal channel by adding media slowly and in rapid succession between wells to keep the volumes between interconnected wells equilibrated. These media volumes were kept constant throughout the experiment.

**Supplementary Figure Legends**

Figure S1: Cultures of neonatal and adult DRG neurons thrive in MFCs. (A) DRG neurons from neonate rats can be seeded in the somal compartment (left) and axons isolated through crossing microgrooves. Axonal outgrowth is evident as early as 24 hrs post-plating (middle), while more extended outgrowth is observed after 6 days in vitro (right). (B) Axonal crossing can be monitored in real time via fluorescent tracer, DiO, applied to the axonal chamber. The dye was taken up by the axons and then retrogradely transported to the cell soma. (C) A microfulidic culture of adult mouse DRG, stained for small sensory neuron marker peripherin. Scale bars = 50 μm.

Figure S2: Phenotypic characterization of DRG cultures in MFCs. (A) DRG neurons in MFC express sodium channels on the cell body (left), axonal segments (middle) and terminals (right). (B) The majority of crossing sensory neurons express CGRP or IB4 markers of nociceptive neurons. (C) A minority of neurons are immunoreactive for the large neuron marker NF200 (arrow). Scale bars = 50μm.

Figure S3: Example of axonal stimulation evoked responses in somal compartment blocked by lidocaine in a 6 well MFC configuration. Images are heat maps representing F340/F380 intensity ratios in Fura-2 loaded neurons (see methods). Arrows indicate neuronal somata responding to axonal stimulation by capsaicin in the distal compartment (A, B). The axonal responses are blocked by addition of lidocaine in the proximal axonal compartment (C). The responses recover after washout (D). See Figure 2 for quantification.

Figure S4: Example axonal capsaicin responses in two MFCs after 48 hr in which the axons were treated with either NGF or anti-NGF antibodies. The soma compartment was treated with anti-NGF antibodies (see methods). B-D and F-H show F340/F380 intensity ratios in Fura-2 loaded neurons. A and E show DiO-positive neurons in the field. B and F show basal levels of Fura-2 340/380 ratio prior to stimulation. Arrows indicate neuronal somata responding to axonal stimulation by capsaicin in control (B, C) and NGF treated (F, J) cultures. D and H represent responses to somal application of 30 mM KCl. See Figure 3 for quantification.

Figure S5: Example of responses to stimulation of axotomized mouse DRG axons in MFCs 72 hrs post axotomy. Images show before (basal) and after axonal stimulation levels of F340/F380 intensity ratios in Fura-2 loaded neurons.

Figure S1


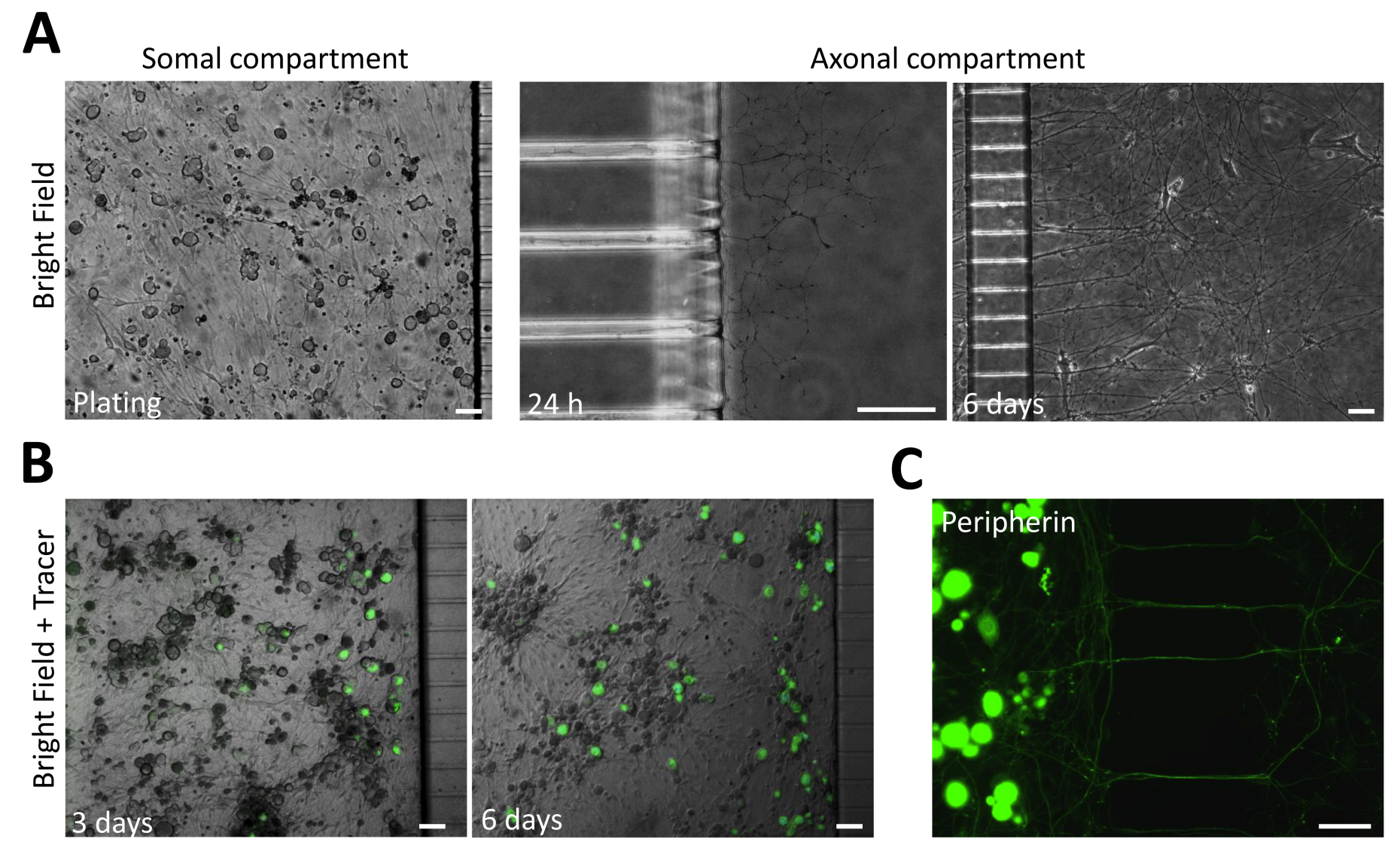


Figure S2


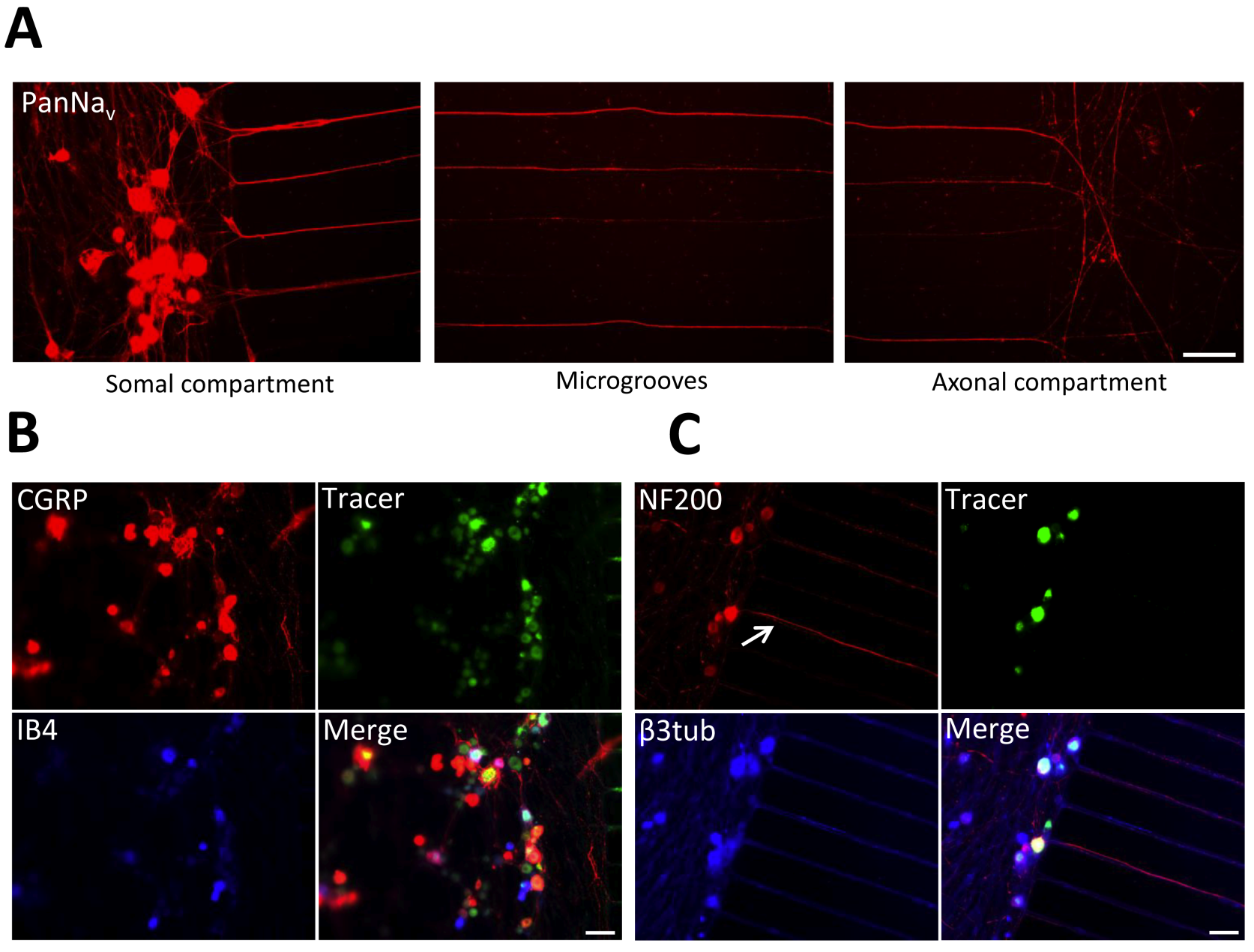


Figure S3


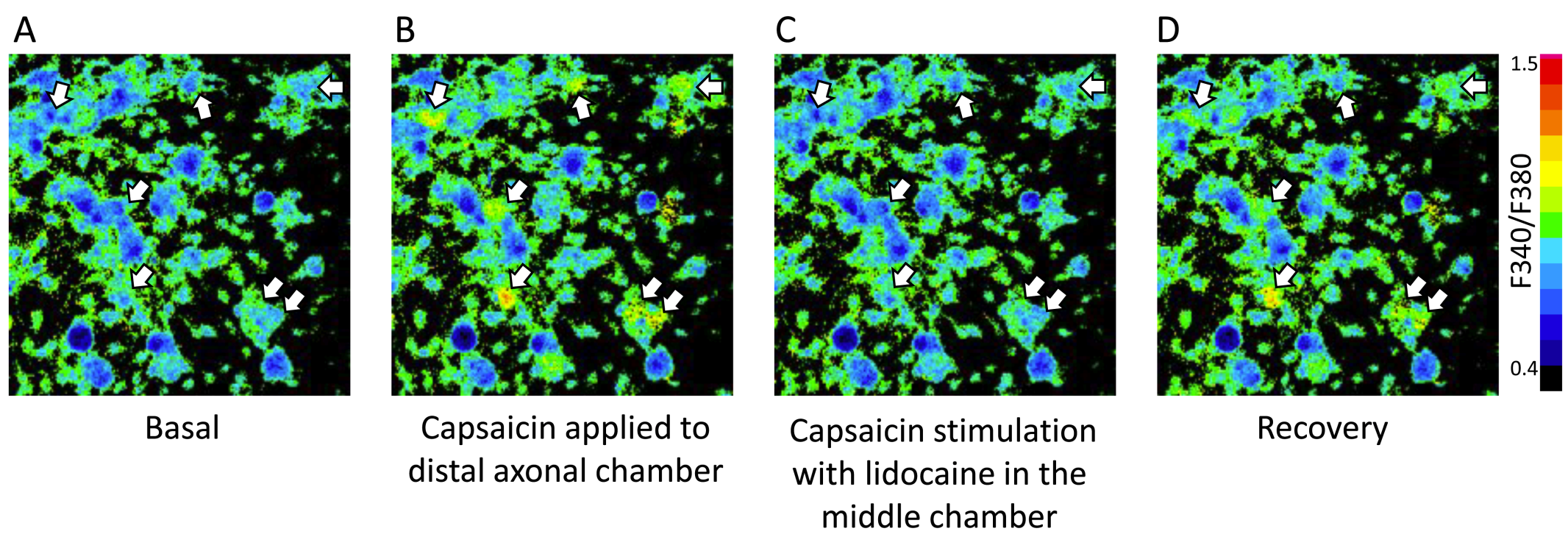


Figure S4


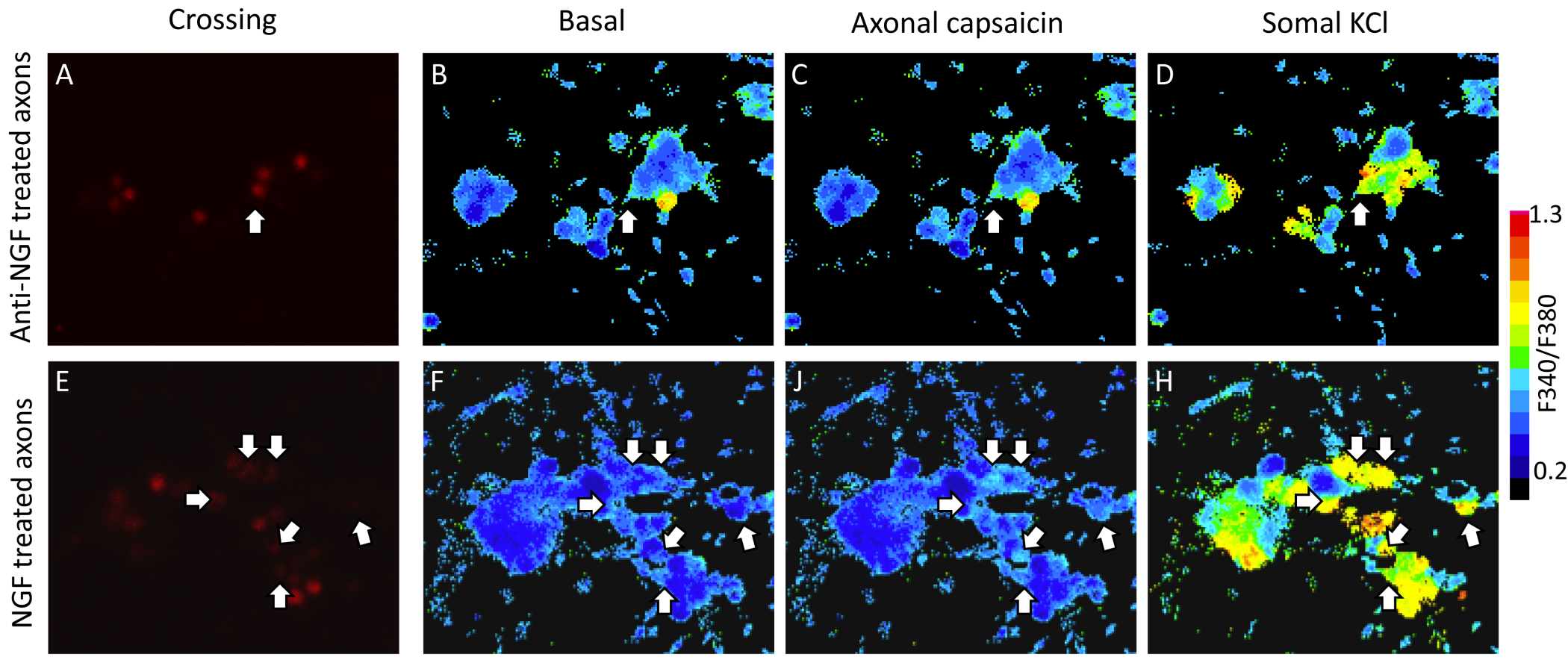


Figure S5


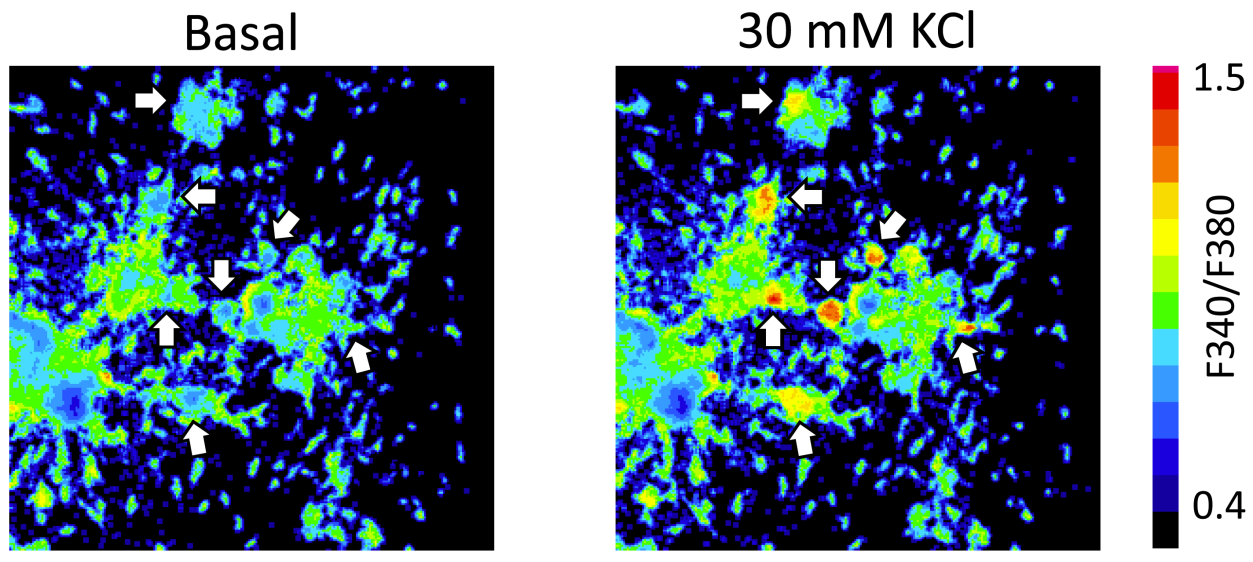

Supplement: File S1 — Supplementary Information. Figure S1, Cultures of neonatal and adult DRG neurons thrive in MFCs. Figure S2, Phenotypic characterization of DRG cultures in MFCs. Figure S3, Example of axonal stimulation evoked responses in somal compartment blocked by lidocaine in a 6 well MFC configuration. Figure S4, Example axonal capsaicin responses in two MFCs after 48 hr in which the axons were treated with either NGF or anti-NGF antibodies. Figure S5, Example of responses to stimulation of axotomized mouse DRG axons in MFCs 72 hrs post axotomy. Methods S1. Results S1. (DOCX) [file pone.0080722.s001.docx]
